# Validation of Quantum Chemistry Predictions. Quinoidal-Base Tautomers in Anthocyanins and Related Compounds

**DOI:** 10.1021/acs.joc.6c01122

**Published:** 2026-07-09

**Authors:** Rui Pereira, Hanieh Mahmoodi, Nuno Basílio, João C. Lima, Mani Outis, Victor De Freitas, Luis Cruz, Fernando Pina

**Affiliations:** † LAQV—REQUIMTE, Departamento de Química e Bioquímica, Faculdade de Ciências, 26706 Universidade do Porto, Rua do Campo Alegre, 687, 4169-007 Porto, Portugal; ‡ LAQV-REQUIMTE, Departamento de Química, Faculdade de Ciências e Tecnologia, 50106 Universidade Nova de Lisboa, 2829-516 Caparica, Portugal; § Centro de Química Estrutural Institute of Molecular Sciences, Instituto Superior Técnico, 225200 Universidade de Lisboa, Lisboa 1649-004, Portugal

## Abstract

Using suitable monoprotic model compounds, we deduce the relative mole fraction distribution of tautomers in anthocyanins and related polyprotic molecules. For species bearing OH at positions C7 and C4′, only the C7 tautomer is relevant. When OH groups occupy positions C7, C5, and C4′, the C7 tautomer is the major with C5 as the minor. The apigeninidin absorption spectrum is reproduced by a model comprising the tautomers with OH at C7 (78%), C5 (21%), and C4′ (1%).

## Graphical abstract

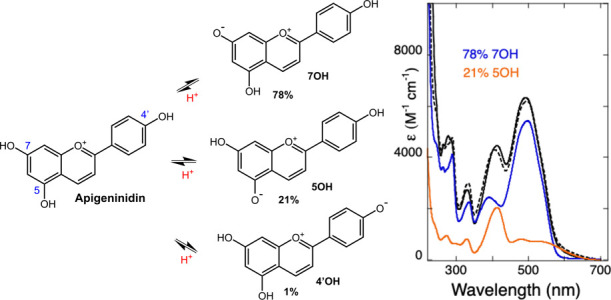

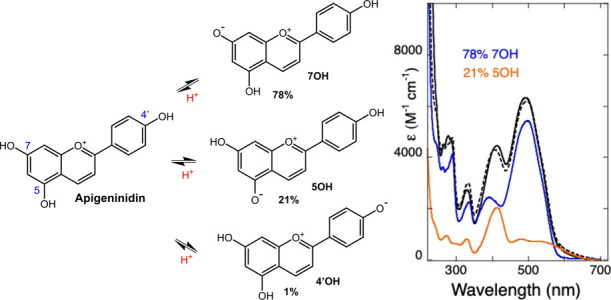

Natural anthocyanin colors can be modulated by quinoidal-base tautomers that have distinct spectral signatures, depending on their relative populations. Because tautomer interconversion is very fast, direct speciation remains elusive. Quantum chemistry offers predictive insights by computing electronic excitations and their intensities, forecasting absorption features and environment-dependent shifts; however, experimental validation is essential. Previous computations dismissed the spectral contribution of the tautomer deprotonated at position 5 and favored those deprotonated at positions 7 and 4′; this conclusion is challenged here.^1^

Hayashi and Ujihara’s theoretical investigation into the color properties of anthocyanidin quinoidal bases offers another compelling applicative example.^2^ Their work provides important computational insights but acknowledges a limitation in experimental validation, citing the difficulty of isolating individual species due to complex dynamic equilibria in aqueous solution: “Unfortunately, these cannot be verified by chemical experiments because it is impossible to prepare a solution consisting of the monomers of each molecular species alone due to the chemical equilibrium of Figure 1.See Supporting Information, Scheme S1, for more details on these author’s Figure 1.

**Figure 1:**
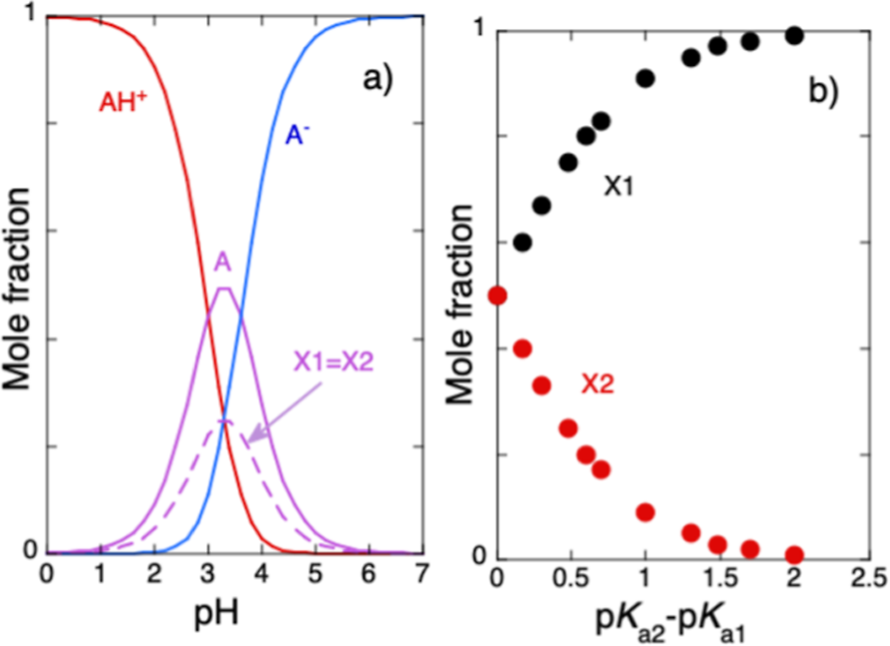
(a) Mole fraction distribution of the tautomers in the case of a stochastic situation; the two tautomers have the same mole fraction distribution (b) Mole fraction distribution of the quinoidal base tautomers as a function of p*K*2 – p*K*1 = log­(n1), eq 6.

Building on these observations, we recently reported in this journal that, contrary to the concerns raised by Hayashi and Ujihara, the absorption spectra of all species in the network of anthocyanins and related compounds can be obtained across the pH range from acidic to neutral solutions using stopped-flow experiments.^3^

In this note, we tackle a remaining open question posed by these authors and, for the first time, provide direct access to the mole fraction of the quinoidal-base tautomers.

Anthocyanins are localized in plant vacuoles, where they contribute to the red-to-blue colors observed in many plants. Here we focus on pH values ranging from acidic to neutral, the range at which vacuolar conditions prevail. Although anthocyanins are characterized by their flavylium cation forms, they comprise a network of interconverting species that reversibly transform through four reactions: proton transfer, hydration, tautomerization, and isomerization, as shown in Scheme 1.^4^

**Scheme 1:**
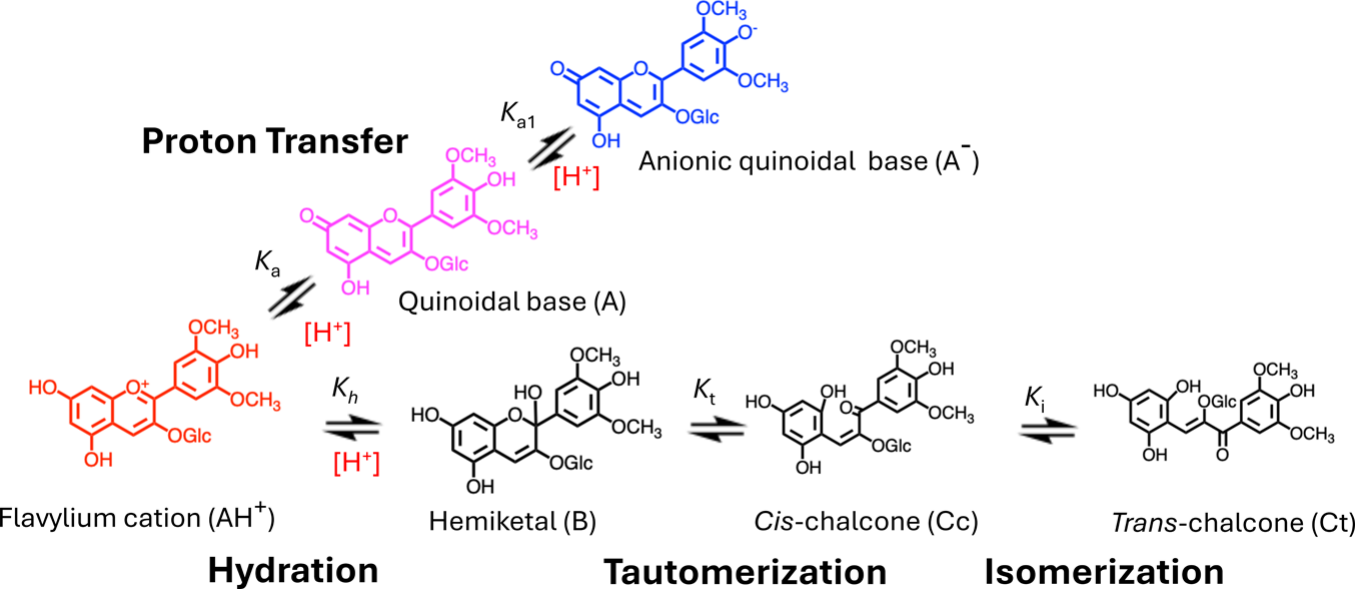
Network of Species Observed in Anthocyanins and Related Compounds in Acidic to Neutral Solutions

Colors arise from the flavylium cation and from neutral and anionic quinoidal bases, Scheme 1. While the flavylium cation is stable in very acidic media, it hydrates at moderately acidic pH values, yielding colorless hemiketal (the major species) or pale yellow *cis*- and *trans*-chalcones (minor species) in simpler anthocyanins. As Brouillard and Dubois demonstrated, the quinoidal bases do not hydrate,^5^ so color persistence depends on shielding the C-2 position of the flavylium cation from water attack, to prevent its hydration.

A crucial question concerns the existence of tautomers for the neutral quinoidal base and of resonance forms for the anionic quinoidal bases, as shown in Scheme 2 for malvidin 3,5-*O*-diglucoside. In other words, the neutral quinoidal base can exist as multiple tautomers that contribute to color, whereas the anionic base has only a single chromophore. Because each tautomer possesses a different absorption spectrum, the observed color is modulated by the relative abundances of the tautomers. The tautomers exist in a rapid equilibrium and cannot be isolated; consequently, some quantum-mechanical studies to overcome this drawback have been reported.^1,2,6^ Here we report experimental evidence for the existence of a single tautomer at position 7 in flavylium compounds bearing two hydroxyl groups in positions 4′ and 7, whereas flavylium compounds bearing three hydroxyl groups exhibit two tautomers at positions 7 (major) and 5 (minor). In both cases, the tautomer at position 4′ can be neglected.

**Scheme 2:**
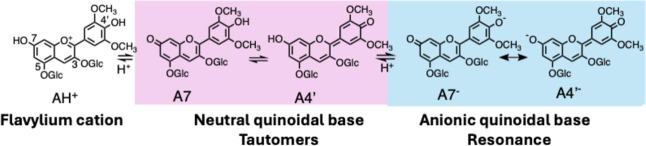
Equilibrium between the Flavylium Cation and the Quinoidal Base Tautomers of Malvidin 3,5-*O*-diglucoside As Well as Resonance Structures of the Anionic Quinoidal Base

In the case of two hydroxyl substituents in position 7 and 4′, we approached this question using a strategy based on our experience with bis-flavylium compounds.^7^ In Scheme 3 the two possible routes for pH dependent equilibrium involving flavylium cation, quinoidal base and anionic quinoidal base are represented in accordance with Scheme 2, i.e. one flavylium cation, two quinoidal base tautomers and one anionic quinoidal base.

$${AH}^{+}+H_{2}O⇌A+H_{3}O^{+}{,K}_{a1}$$ 1

$$A+H_{2}O⇌A^{-}+H_{3}O^{+}{,K}_{a2}$$ 2

It is straightforwardly to demonstrate that eq 1 can be expressed in terms of the micro equilibrium constants *K*1 and *K*2, eq 3

3 $$K_{a1}=K1+K2$$

and the second equilibrium, eq 2, in terms of *K*3 and *K*4, eq 4. See Supporting Information, Section S2.

4 $$K_{a2}=\frac{K3K4}{K3+K4}$$

In a previous study,^7^ we demonstrated that if the bridge of a bis-flavylium compound prevents electronic communication or any through–space interaction between the two terminals, the system enters a stochastic regime. All equilibrium constants are equal, and eq 5 is satisfied, see Supporting Information, Section S2.

5 $$K_{a1}/K_{a2}=4\;\text{and}\;pK_{a2}-pK_{a1}=\log\left(4\right)$$

**Scheme 3:**
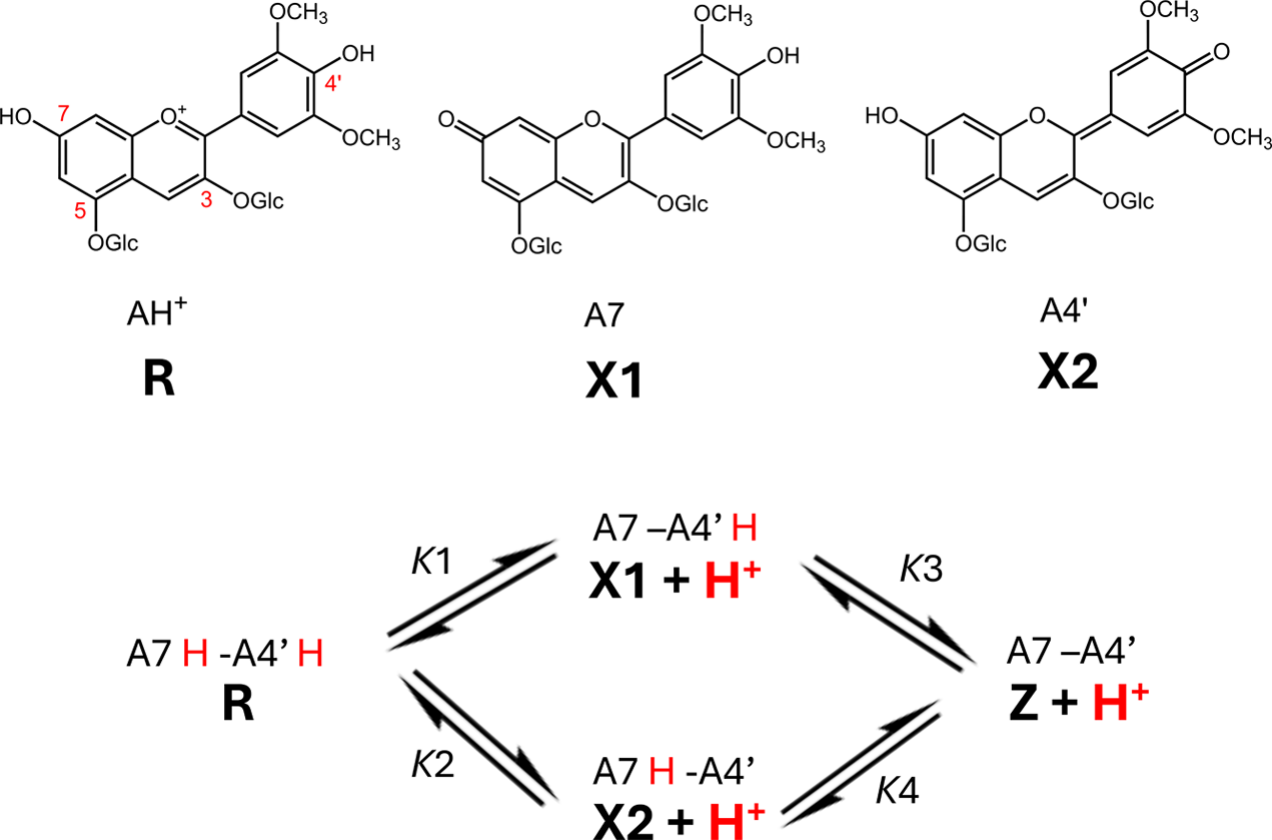
Malvidin, 3,5-*O*-Diglucoside Tautomers of Quinoidal Base^a^

a This scheme can be applied for all flavylium compounds bearing two hydroxyl substituents in position 7 and 4′.

For the present system, one can conceive a similar (hypothetical) scenario in which positions 7 and 4′ have equal acid–base constants. This situation corresponds to Figure 1a.

When the two p*K*_a_’s are different the parameter n1 could be defined

6 K1=n1K2log(n1)=pK2−pK1

and from the relation *K*1*K*3 = *K*2*K*4

7 K4=n1K3

n1 regards the ratio between the acidity constant in position 7 and position 4′. For n1 > 1, the acidity constant in position 7 is higher than in position 4′ by n1.

Substituting eqs 6 and 7 in eqs 3 and 4

8 $$K_{a1}=\left(1+n1\right)K2$$

9 $$K_{a2}=\frac{n1K3}{\left(1+n1\right)}$$

For each value of n1, *K*2 and *K*3 are obtained respectively from eqs 8 and 9 and K1 and K4 from eqs 6 and 7.

In conclusion, from the experimentally obtained p*K*_a_ values, all micro acidity constants can be obtained as a function of log­(n1), as well as the mole fraction of the tautomers X1 and X2, Figure 1b, X1 = *K*1/(*K*1 + *K*2) and X2 = *K*2/(*K*1 + *K*2).

The remaining question is how to estimate the value of n1 to be used in Figure 1b, in order to obtain the mole fractions of the corresponding tautomers at positions 7 and 4′.

Resuming to Scheme 3, the molecule either follows *K*1*K*3 path or *K*2*K*4 path, i.e., there are independent paths. As such the values of 7-hydroxyflavylium p*K*_a_ = 3.55,^8^ and of 4′-hydroxyflavylium p*K*_a_ = 5.5 ^9,10^ have been used as reasonable estimates for *K*1 and *K*2, yielding log­(n1) = 1.95, (n1 = 89) corresponding to X1 = 99% and X2 = 1% in Figure 1b. A similar calculation can be made for other related compounds monohydroxylated at positions 7 and 4’.^11^ In this case p*K*_a_ = 1.7 (n1 = 50), X1 = 98% and X2 = 2%.

It can be concluded that for the neutral quinoidal base of malvidin-3,5-*O*-diglucoside and similar compounds only tautomer deprotonated at 7, X1 in Scheme 3, should be considered, since the one in 4′ is negligible. This conclusion is consistent with the long-standing observation by Jurd and Geissman (1963) that in 4′,7-dihydroxyflavylium the tautomer 7 is the preferential structure.^12^

To analyze malvidin-3-*O*-glucoside (trihydroxylated at 7, 5, 4′), the model with an additional acid–base constant and update Scheme 3 (Scheme S4, Supporting Information) is required. While experimental evidence supports the deprotonation sequence at positions 7 and 4′ in malvidin-3,5-diglucoside, based on the differences in p*K*_a_ found for 7-hydroxyflavylium and of 4′-hydroxyflavylium, such evidence is lacking for the sequence adopted for malvidin-3-*O*-glucoside, particularly regarding the relative deprotonation magnitude between positions 5 and 7. This represents the first limitation of the analysis, which would be overcome by the study of the compound 5-hydroxyflavylium if it were not very unstable^13^

Nevertheless, an analysis is possible with a minimum of assumptions. While the three macroscopic acid base constants of malvidin 3-*O*-glucoside could be used, for our scope only *K*_a1_, is required.

The first acid base constant *K*_a1_ is now given by eq 10, and its experimental value is coincident to the one of malvidin 3,5-*O*-diglucoside.^14^

10 $$K_{a1}=\frac{\left(\left[X1\right]+\left[X2\right]+\left[X3\right]\right)\left[H^{+}\right]}{\left[R\right]}=K1+K2+K3{=10}^{-3.8}$$

Introducing the parameters n1 and n2, with n1 previously defined in eq 6 and n2 in eq 11

K1=n1K2andK2=n2K3⁢ log(n2)=pK3−pK2 11

Substituting eqs 11 in [Disp-formula eq10] and taking the experimental value of p*K*_a1_ = 3.8

12 $$\left(n1n2+n2+1\right)K3=10^{-3.8}$$

From eq 13, the ratio K1/K3 can be calculated

13 K1/K3=n1n2

An assumption of the system is to consider that the difference between deprotonation in position 7­(*K*1) and 4′(*K*3) in the case of three hydroxyl substituents can be approximated as in the case of malvidin 3,5-*O*-diglucoside (*K*1/*K*2 = 89), leading to the same value for K1/K3 in malvidin 3-*O*-glucoside. From eq 13

14 K1/K3=89=n1n2

Substitution of eqs 14 in [Disp-formula eq12], leads to eq 15

15 $$\left(89+n2+1\right)K3=10^{-3.8}$$

For each n2, a value of *K*3 is obtained from eq 15, a value of *K*2 by eq 11 and a value *K*1 from eq 10.

Having the microequilibrium constants of each tautomer the mole fraction of the 3 tautomers is directly calculated (e.g., X1 = *K*1/(*K*1 + *K*2 + *K*3)), Figure 2, based on the single assumption that 7-hydroxyflavylium and of 4′-hydroxyflavylium are adequate models to predict deprotonation for 7 and 4′ positions.

**Figure 2:**
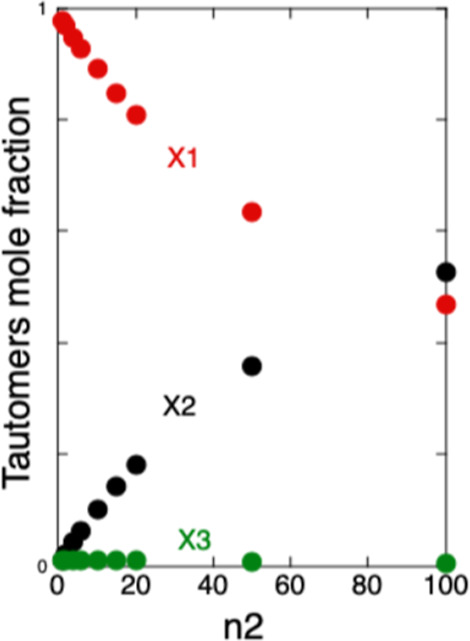
Representation of the mole fractions of the 3 tautomers of malvidin 3-*O*-glucoside versus n2. This figure is also valid for apigeninidin, see below.

A straightforward generalization is that deprotonation in 4′ is always negligible with respect to 7. The weight of the deprotonation in position 5 depends on the value of n2. The question is to find the model compounds to estimate n2.

An approximation to account for the relative value of the acidity constants at positions 7, 5 and 4′ is to make the calculations for apigeninidin (deprotonations at 7, 5, and 4′ positions) and other 3 model compounds of 3-deoxyanthocyanins, each one able to deprotonate at only one of the positions, 7, 5 or 4′, Scheme 4. The model compounds reported in Scheme 3 were prepared for this purpose and the absorption spectra versus pH (flavylium cation and neutral quinoidal bases) collected, see Supporting Information for the details of their synthesis characterization and absorption spectra (Section S3–S5).

**Scheme 4:**
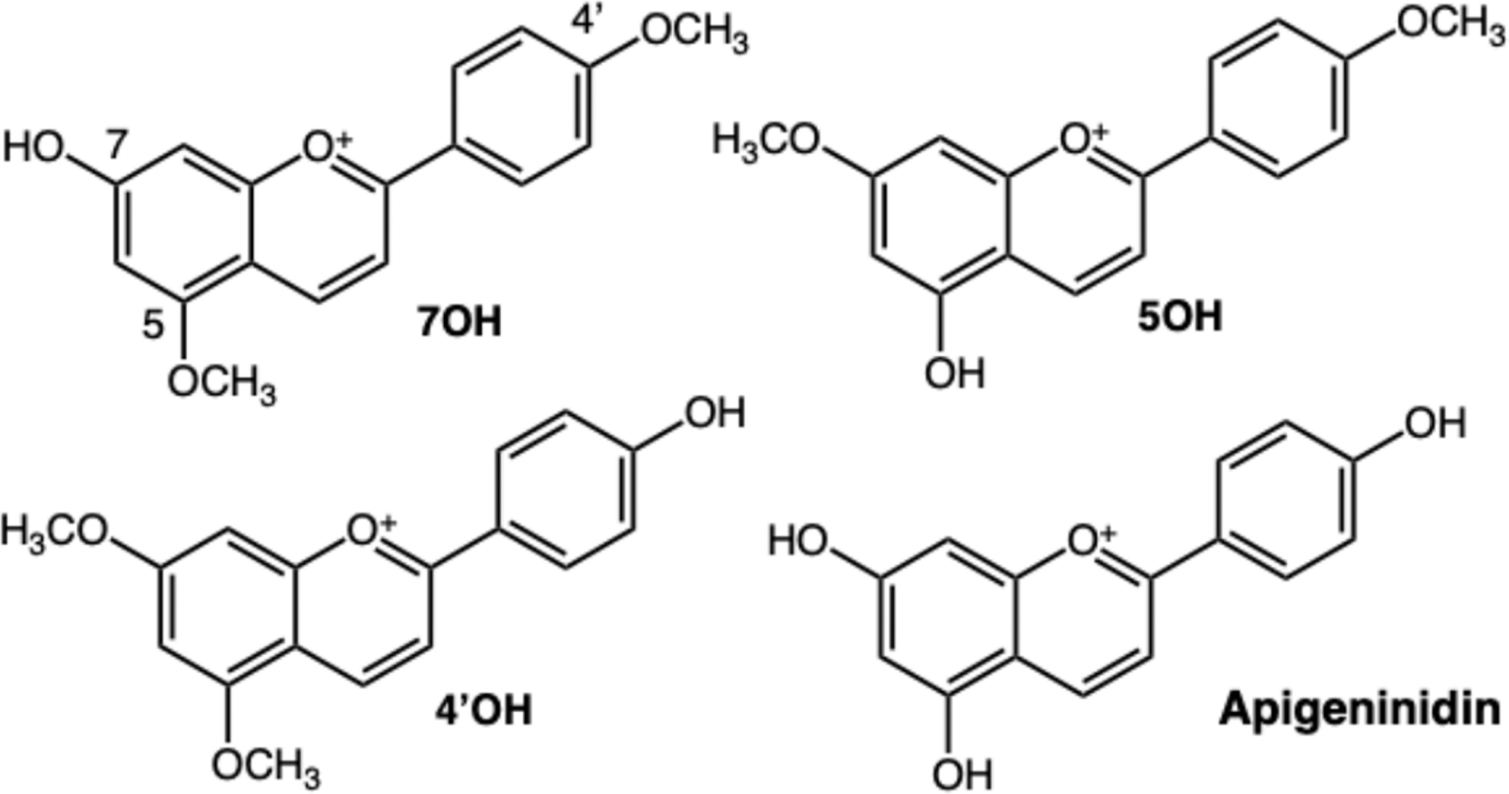
Apigeninidin and the Synthesized Model Compounds^a^

a See Supporting Information for the details of the synthesis and characterization.

The absorption spectra of the neutral quinoidal bases selected at a pH where there is a minimal interference of the flavylium cation and the anionic quinoidal base were converted in ε (mole absorption coefficients) and used to fit the absorption spectra of apigeninidin, with the model compounds of Scheme 3, Figure 3b.

**Figure 3:**
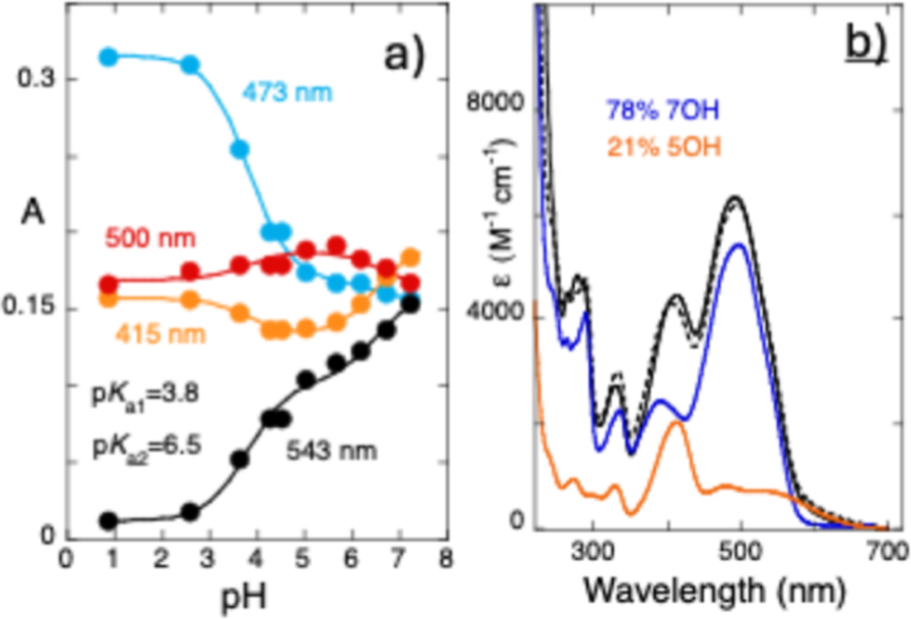
(a) Absorbance at selected wavelengths used to calculate the acid base constants of eqs 1 and 2. (b) Fitting of the apigeninidin quinoidal base absorption spectrum with tautomer 7OH and tautomer 5OH.

Since the first acid base constant for apigeninidin is also p*K*_a1_ = 3.8, Figure 2 deduced for malvidin 3-*O*- glucoside is also valid for apigeninidin. Otherwise in eq 12 the p*K*_a1_ would have to be modified.

An important breakthrough arouses from the observation that the absorption spectra of apigeninidin quinoidal base can be fitted with a linear combination of the quinoidal bases of the monohydroxylated analogues. This allowed us to achieve an adequate fit to the absorption spectrum of the neutral-base apigeninidin and retrieve the composition of the tautomer mixture, consisting of 78% of the 7OH absorption spectrum, 21% of the 5OH spectrum, and 1% of the 4′OH spectrum, Figure 3b. These tautomers fraction gives an estimate of n2 = 15.4 for the case of apigeninidin in Figure 3. Having the n2 value the acid base equilibrium constants can be calculated (eqs 15, 11 and 10), Table 1. In this table, an error of ±4 on the n2 value leads to an error of ±0.07 for X1, ±0.07 for X2 and ±0.01 for X3. Clearly tautomer 7 is the major one, followed by tautomer 5 and tautomer 4′ is residual.

**Table 1:** Prediction of the Tautomers Mole Fraction of Apigeninidin (n2 = 15.4)

X1	X2	X3
0.78(A7)	0.21(A5)	0.01(A4′)
*K*1	*K*2	*K*3
1.2 × 10^–4^	3.3 × 10^–5^	1.3 × 10^–6^

It is important to stress that based on the experimental values of Δp*K*_a_ for positions 4′ and 7 retrieved from compounds 7-hydroxyflavylium and of 4′-hydroxyflavylium (1.95), other monohydroxylated derivatives (1.7),^11^ and from 7OH and 4′OH compounds in Scheme 4 (1.9), see Supporting Information Figure S4, the use of model compounds to reproduce the order of deprotonation and the differences in p*K*_a_, for the different positions, seem to be adequate, since the substitution pattern has only moderate effects on the values, which are within the experimental error in p*K*_a_ determination. In conclusion, apigeninidin’s quinoidal-base mole fractions provide good estimates for malvidin 3-*O*-glucoside.

The color of anthocyanins at moderately acidic pH values is largely defined by the relative populations of their quinoidal base tautomers, which reshape electronic transitions and thus the observed hue. While in the case of two hydroxyl units there is evidence for the existence of a single tautomer, for there hydroxyl units the tautomer in position 5 needs to be considered. In the case of more than 3 hydroxyl substituents, the acid–base p*K*_a_s tend to be closer and more quinoidal base tautomers are expected to contribute to the definition of the quinoidal base color.

To confirm the relative acidity at 7OH, 5OH and 4́OH positions in the model molecules, we have adapted a Quantum Chemical methodology reported in the literature where the relative acidities of OH groups can be inferred by comparing the relative change in the energy as the O–H distance is increased.^15^ By converting the calculated energy difference into the dimensionless Δ*E* = Δ*E* (in eV)/0.059 V, a trend can be observed which asymptotically approaches Δp*K*_a_. The calculated Δp*K*_a_ values for the monohydroxylated compounds in Scheme 4, correctly reproduce the order of deprotonation obtained experimentally (Figure S25). The same computational methodology was applied to apigeninidin revealing the same relative acidity at 7OH, 5OH and 4́OH positions (Figure S26).

Quantum-chemical predictions for anthocyanins and related compounds can be validated, and at least some of their limitations can be assessed within a validation framework.

## Supplementary Material



## Data Availability

The data underlying this study are available in the published article and its Supporting Information.

## Notes

Additional details on the kinetics and thermodynamics of the pH-dependent equilibria in the anthocyanin reaction network. Microscopic and apparent acid–base equilibrium constants of diprotic and triprotic compounds. pH-dependent UV–vis spectra and interconversion kinetics of model compounds. Experimental procedures for the synthesis of model compounds. ^1^H, ^13^C, HBMC, HSQC NMR spectra. Results of DFT calculations, including simulated UV–vis spectra and evaluation of Δp*K*_a_ (PDF)
