# Supplementary material for: Validation of Quantum Chemistry Predictions. Quinoidal-Base Tautomers in Anthocyanins and Related Compounds
Source: J Org Chem. 2026 Jul 9;91(29):10336–41. doi: 10.1021/acs.joc.6c01122 (PMC13411063; doi:10.1021/acs.joc.6c01122)
Supplement: Supplementary file 1 [file jo6c01122_si_001.pdf]

Supporting information

Validation of Quantum Chemistry Predictions. Quinoidal-base Tautomers in Anthocyanins and Related Compounds.

Rui Pereira[a†] Hanieh Mahmoodi [b†] Nuno Basílio[* b] João C. Lima [b] Mani Outis [b] Victor de Freitas [a] Luis Cruz[*a] Fernando Pina[b]

LAQV – REQUIMTE, Departamento de Química e Bioquímica, Faculdade de Ciências, Universidade do Porto, Rua do Campo Alegre, 687, 4169-007 Porto, Portugal

LAQV – REQUIMTE, Departamento de Química, Faculdade de Ciências e Tecnologia, Universidade Nova de Lisboa, 2829-516 Caparica, Portugal, fp@fct.unl.pt

Table of contents

1. More details on Hayashi and Ujihar Fig. 1 and reverse pH jumps S2

2. Micro equilibrium acid-base constants S5

2.1. Extension to 3 hydroxyl substituents S6

3. Synthesis and characterization S7

3.2. Synthesis S7

Synthetic Benzaldehydes **1-3** S7

Flavylium dyes **a-c** S8

4. Spectroscopic measurements S10

5. NMR. Spectra S12

6. Computational studies S27

6.1. TDDFT simulated spectra S27

6.2. ΔpK_a evaluation S28

6.3. Cartesian coordinates S29

6.4. Absolute energies S45

References S47

1. More details on Hayashi and Ujihar Fig.1 and reverse pH jumps

Figure 1 of Hayashi and Ujihar, regards the well-known thermodynamic and kinetic Scheme presented in more detail in Scheme S1.[1] In this scheme the free Gibbs energy of the five network species are presented versus pH. Flavylium cation is the sole species at very acidic medium. After addition of base (direct pH jumps), for example pH=6, it is no longer the more stable species. Quinoidal base is formed initially because proton transfer is by far the faster reaction of the network. Brouillard and Dubois[2] discovered that in acidic medium the quinoidal base is stable and that the system evolves toward the equilibrium via hydration of the flavylium cation followed by a faster tautomerization reaction, leading to a kinetic process controlled by the hydration. The equilibrium is reached through the much slower *cis-trans* isomerization.

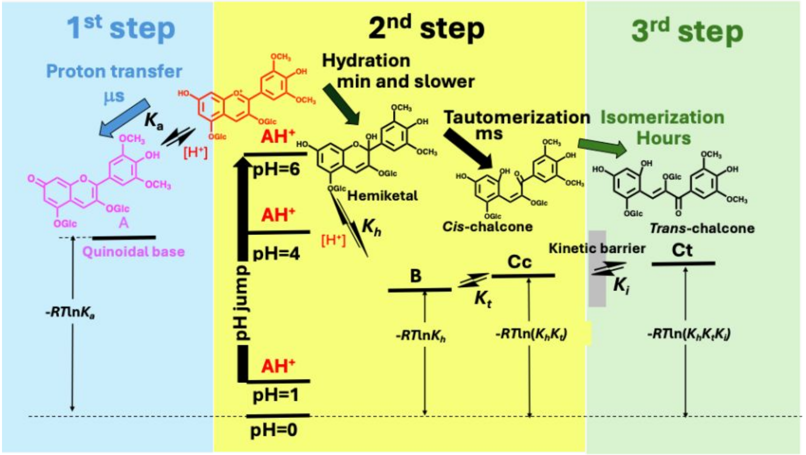

Scheme S1. Relative energy level diagram of malvidin-3,5-*O*-diglucoside (Malvin), after a pH jump to pH=6. A similar diagram is obtained for the other anthocyanins. In the quinoidal base form, deprotonation is most likely to occur at position 7; however, the contribution of other tautomeric forms cannot be ruled out, see below.

The sequence of reactions of Scheme 1 is described by eq.1 through eq.4:

$AH^+ + H_2O \rightleftharpoons A + H_3O^+$ K_{a1} Proton transfer (1)

$AH^+ + H_2O \rightleftharpoons B + H_3O^+$ K_h Hydration (2)

$B \rightleftharpoons Cc$ K_t Tautomerization (3)

$Cc \rightleftharpoons Ct$ K_i Isomerization (4)

A significant simplification of this reaction sequence can be achieved by considering a single acid-base equilibrium between the flavylium cation and its conjugate base, CB, as shown in equation (5)[3,4]

$AH^+ + H_2O \rightleftharpoons CB + H_3O^+$ $\quad K'_a \quad$ global equilibrium (5)

$[CB]=[A] + [B] + [Cc] + [Ct]$ (6)

And $\quad K'_a = K_a + K_h + K_hK_t + K_hK_tK_i$ (7)

The mole fraction distribution of the stopped flow species can be given by the pseudo-equilibrium that includes all species except trans-chalcone and is defined by the symbol (^).

$AH^+ + H_2O \rightleftharpoons CB + H_3O^+$ $\quad K^\wedge_a \quad$ global equilibrium (9)

$[CB^\wedge]=[A] + [B] + [Cc]$ (10)

$$K^\wedge_a = K_a + K_h + K_hK_t$$

The construction of Scheme 1 requires determination of the four equilibrium constants. This is straightforwardly achieved by carrying out reverse pH jumps defined by addition of acid to equilibrated solutions at higher pH values. In supplementary information a detailed description of the equilibrium constants calculation as well as the construction of the energy level diagram are presented.

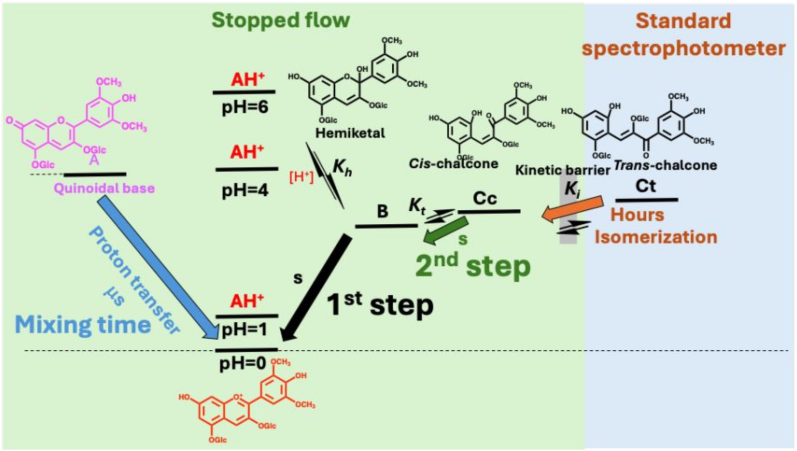

Scheme S2. Reverse pH jumps in malvidin 3,5-*O*-diglucoside (Malvin).

The kinetic processes upon a reverse pH jump in anthocyanins and flavylium based systems exhibiting high activation energy for the cis-trans isomerization are summarized in Scheme S2 and Fig. S1.

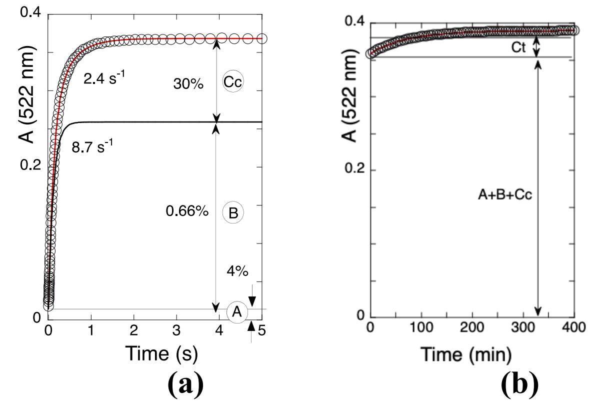

Figure S1. (a) Reverse pH jumps of Malvidin 3,5-O-diglucoside (Malvin) at pH=4.2 to [HCl]=0.5 M, monitored by stopped-flow; **(b**) The same of a) monitored by a standard spectrophotometer.

The stopped flow trace is bi-exponential and there is an initial absorbance, see Fig.S1a. The initial absorbance occurs during the mixing time of the stopped flow and corresponds to the conversion of quinoidal base in flavylium cation together with some flavylium cation that could be present before the reverse pH jump at more acidic solutions (at pH=4.2 the mole fraction of flavylium cation is negligible). The first kinetic step is due to the conversion of hemiketal in more flavylium cation, because at very acidic pH values the hydration (more properly the dehydration) becomes faster than tautomerization, the so-called change of regime.[5] The last step of the stopped flow (second step in Scheme 2) corresponds to the formation of more flavylium cation form cis-chalcone via hemiketal.[6] Consequently, the three amplitudes correspond to the fractions of the species A, B and Cc at the initial pH 4.2. Normalization to the unity gives the mole fractions of the respective species and extension to all pH range the respective mole fraction distribution of these species at the pseudo-equilibrium, from which the equilibrium constants of eq. 1 to eq.3 can be calculated, see supplementary material. The calculation of the equilibrium constant of eq.4 requires a stopped flow followed by a standard spectrophotometer, see Fig.S1b. The respective trace results from the slow conversion of trans-chalcone in flavylium cation. The initial amplitude regards the flavylium cation formed initial (it takes *circa* 1 min to take the first absorption spectrum) form all species except Ct, and the amplitude of the kinetic step is proportional to the fraction of

this last species. Normalization and extension to all pH range, permits to obtain the mole fraction distribution of trans-chalcone and the respective equilibrium constant.

2. Micro equilibrium acid-base constants

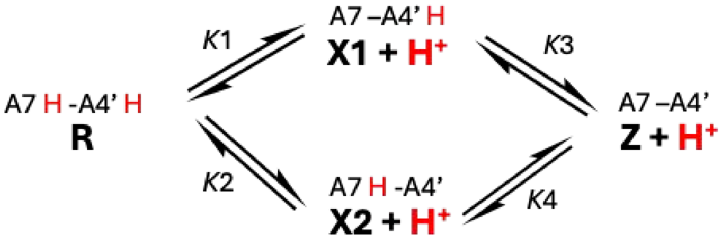

Scheme S3

According to the Scheme 1 the following micro-acid base equilibria can be defined: R=AH^+, X1 and X2 the tautomers of the neutral quinoidal base and Z=A^- the anionic quinoidal base.

$K1 = \frac{[X1][H^+]}{R}$; $K2 = \frac{[X2][H^+]}{R}$; $K3 = \frac{[Z][H^+]}{[X1]}$; $K4 = \frac{[Z][H^+]}{[X2]}$

Defining eq.1 and using the definition of $K1$ and $K2$

$$AH^+ + H_2O \rightleftharpoons A + H_3O^+ \qquad K_{a1} \qquad (1)$$

$$K_{a1} = \frac{([X1]+[X2])[H^+]}{R} = K1+K2 \qquad (2)$$

Defining eq.3 and using the definitions of $K3$ and $K4$

$$A + H_2O \rightleftharpoons A^- + H_3O^+ \qquad K_{a2} \qquad (3)$$

$$K_{a2} = \frac{[Z][H^+]}{[X1]+[X2]} = \frac{[Z][H^+]}{\frac{[Z][H^+]}{K3}+\frac{[Z][H^+]}{K4}} = \frac{1}{\frac{1}{K3}+\frac{1}{K4}} = \frac{K3K4}{K3+K4} \qquad (4)$$

Quod erat demonstrandum

Stochastic situation

In the case of the stochasric approximation all constants are equal (Km), K_{a1}=2Km and K_{a2}=Km/2. Consequently

$$\frac{K_{a1}}{K_{a2}} = 4 \qquad pK_{a2}-pK_{a1}=\log 4=0.6$$

2.1. Extension to 3 hydroxyl substituents

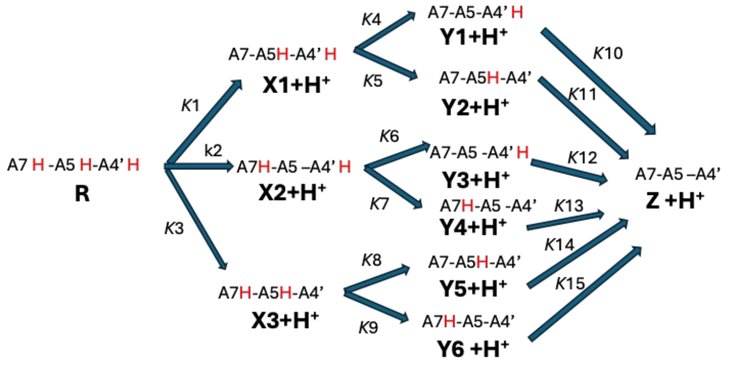

Scheme S4

Scheme 4 is the extension of Scheme 3 for the case of 3 hydroxy substituents.

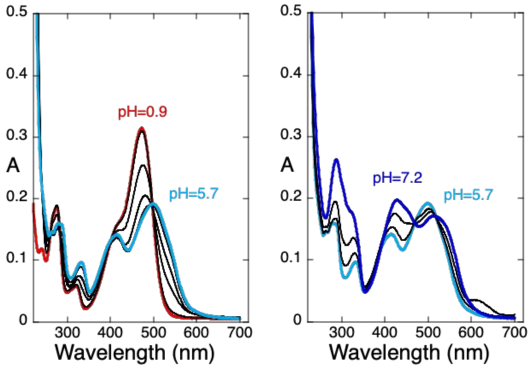

(a) **(b)**

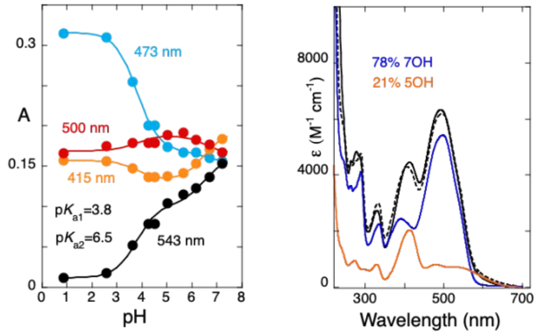

(b) **(c)**

Figure S2. **(a)** pH dependent spectral variations of apigeninidin for 0.9<pH<5.7; **(b)** the same for 5.7<pH<7.2; **(c)** absorbance at selected wavelengths used to calculate the acid base constants of eq.1 and eq.2. **(d)** Fitting of the apigeninidin quinoidal base absorption spectrum with tautomer 7OH and tautomer 5OH.

3. Synthesis and characterization of the model compounds.

3.1. Materials

1-(4-hydroxyphenyl)ethan-1-one, 1-(4-methoxyphenyl)ethan-1-one, 2,4,6-trihydroxybenzaldehyde and trimethylchlorosilane (TMSCl) were purchased from Sigma-Aldrich (Madrid Spain). M3G, M3,5GdiG and Apigeninidin were purchased from Extrasynthese ≥95%. All other chemicals were of analytical grade. pH jumps monitored by UV-Vis and stopped-flow are described elsewhere.[8]

3.2. Synthesis

Synthetic Benzaldehydes **1-3**

Three synthetic benzaldehyde derivatives were first obtained through methylation reactions: 2,4-dihydroxy-6-methoxybenzaldehyde (**1**) and 2,6-dihydroxy-4-methoxybenzaldehyde (**2**) were both synthesized from the monomethyl protection of 2,4,6-trihydroxybenzaldehyde (1.3×10^{-3} mol, 200 mg) with MeI (2 ml) and $KHCO_3$ (144 mg, 1.1 equiv.) in acetone (15 mL) under reflux overnight in a oil bath. 2-hydroxy-4,6-dimethoxybenzaldehyde (**3**) was synthesized from adding 2,4,6-trihydroxybenzaldehyde (1.3×10^{-3} mol, 200 mg) and MeI (2 ml) and K_2CO_3 (280 mg, 3 equiv.) in DMF (10 mL) under reflux overnight in a oil bath. The formation of the protected aldehydes was monitored with by HPLC-DAD, in a reversed-phase C18 column (150 × 4.6 mm i. d., 5 µm, Thermo Scientific).

To stop the reaction, sodium hydroxide solution (20 mL, 1 M) was added, and the mixture was purified through liquid-liquid extraction using dichloromethane. The organic phase was washed with water until neutral pH, dried over anhydrous sodium sulfate, and concentrated under reduced pressure. The residue obtained the stnthesis of **1** and **2** was purified by silica gel chromatography (60 A.p.s, 35-70 µm) with hexane/ethyl acetate (3:1) as eluent, to afford an inseparable mixture of two isomers, 2,4-dihydroxy-6-methoxybenzaldehyde and 2,6-dihydroxy-4-methoxybenzaldehyde. The residue obtained from the synthesis of **3** was purified by silica gel chromatography (60 A.p.s, 35-70 µm) with hexane/ethyl acetate (2:1) as eluent, to afford pure 2-hydroxy-4,6-dimethoxybenzaldehyde.

(**1**) White solid (0.025g, 11 % yield). **^{1}H NMR** (400 MHz, CD_3CN) *δ* 12.40 (s, 1H, C2-OH), 10.07 (s, 1H, CHO), 5.96 (s, 2H, H3, H5), 3.80 (s, 3H, C4-OCH_3). **$^{13}C\{^1H\}$ NMR** (101 MHz, CD_3CN) δ 192.6 (CHO), 169.2 (C4), 164.8 (C2+C6), 106.4 (C1), 94.1 (C3 +C5), 56.5 (OCH_3). **MS** (ESI) *m/z*: $[M-H]^-$ Calcd for $C_8H_8O_4$ 167.0350; Found 167.0349.

(**2**) White solid (0.025g, 11 % yield). **^{1}H NMR** (400 MHz, CD_3CN) *δ* 12.40 (s, 1H, C2-OH), 10.03 (d, *J* = 0.6 Hz, 1H, CHO), 5.98 (d, *J* = 2.1 Hz, 1H, H5), 5.90 (dd, *J* = 2.1, 0.6 Hz, 1H, H3) 3.85 (s, 3H, C6-OCH_3). **$^{13}C\{^1H\}$ NMR** (101 MHz, CD_3CN) δ 192.9 (CHO), 167.5 (C-4), 166.8 (C-2), 165.5 (C6-OCH_3), 106.4 (C1), 96.0 (C3), 91.9 (C5), 56.7 (OCH_3). **MS** (ESI) *m/z*: $[M-H]^-$ Calcd for $C_8H_8O_4$ 167.0350; Found 167.0349.

(**3**) White solid (0.1940g, 82% yield). Known and commercially available. **^{1}H NMR** (400 MHz, $CDCl_3$) *δ* 12.51 (s, 1H, C2-OH), 10.09 (s, 1H, CHO), 6.01 (d, *J* = 2.2 Hz, 1H, H3), 5.91 (d, *J* = 2.2 Hz, 1H, H3), 3.85 (s, 3H, C4-OCH_3), 3.83 (s, 3H, C6-OCH_3). ^{13}C NMR (101 MHz, $CDCl_3$) δ 192.79 (CHO), 169.05 (C4), 167.29 (C2), 164.47 (C6), 106.94 (C1), 93.81 (C3), 91.51 (C5), 56.64 (C4-OCH_3), 56.63 (C6-OCH_3). **$^{13}C\{^1H\}$ NMR** (101 MHz, $CDCl_3$) δ 192.8 (CHO), 169.1 (C2), 167.3 (C4), 164.5 (C6), 106.9 (C1), 93.8 (C3), 91.5 (C5), 56.63 (OCH_3), 56.64 (OCH_3). **MS** (ESI) *m/z*: $[M-H]^-$ Calcd for $C_9H_{10}O_4$ 181.0506; Found 181.0505.

Flavylium dyes **a-c**

Three flavylium dyes, namely 7-hydroxy-4',5-dimethoxyflavylium (compound **a**), 5-hydroxy-4',7-dimethoxyflavylium (compound **b**) and 4'-hydroxy-5,7-dimethoxyflavylium (compound **c**) were obtained from acid-catalyzed aldol condensation reactions between previously obtained synthetic benzaldehydes and two different acetophenones, based on the mechanism already described by Robinson.

Compounds **a** and **b** were synthesized from adding a mixture of 2,4-dihydroxy-6-methoxybenzaldehyde (**1**) (3×10^{-4} mol, 50 mg) and 2,6-dihydroxy-4-methoxybenzaldehyde (**2**), respectively, (3.0×10^{-4} mol, 50 mg) with 4-methoxyacetophenone (6.7×10^{-4} mol, 100 mg); and compound **c** between 2-hydroxy-4,6-dimethoxybenzaldehyde (**3**) (6×10^{-4} mol, 100 mg) and 4-hydroxyacetophenone (6.6×10^{-4} mol, 100 mg). The synthesis of compound **c** was carried out in 3 mL of a mixture of ethyl acetate:methanol (2:1) in the presence of TMSCl (20 equivalents, 600 μL) and left to react at 0 ºC during 2 h, under stirring. All remaining syntheses were carried out at room temperature during 24 h. The formation of the new pigments was monitored by HPLC-DAD, in a reversed-phase C18 column (150 × 4.6 mm i. d., 5 μm, Thermo Scientific) and through LC-MS analysis. To stop the reaction, water (30 mL) was added and then, the mixture was purified

through liquid-liquid extraction using ethyl acetate. The aqueous phase containing mainly the flavylium compounds was later purified by column chromatography using LiChroprep® RP-18 (40–63 μm). Compounds **a** and **b** were both isolated from the same column with increasing percentages of acidified methanol (from 10 % V/V to 40% V/V). All three dyes displayed an orange color. After evaporating methanol at 38 °C, the purified pigments were freeze-dried and kept at −20 °C.

(**a**) Orange solid (0.048g, 56 % yield). **^{1}H NMR** (600 MHz, MeOD) *δ* 8.92 (d, *J* = 8.7 Hz, 1H, H4), 8.18 (d, *J* = 9.1 Hz, 2H, H2’,H6’), 7.95 (d, *J* = 8.7 Hz, 1H, H3), 7.02 (d, *J* = 9.1 Hz, 2H, H3’,H5’), 6.85 (d, *J* = 1.9 Hz, 1H, H8), 6.58 (d, *J* = 1.9 Hz, 1H, H6), 3.87 (s, 3H, OCH_3), 3.75 (s, 3H, OCH_3). **^{13}C{^{1}H} NMR** (151 MHz, MeOD) δ 171.5 (C8a), 171.3 (C2), 166.7 (C4’), 159.8 (C5), 159.1 (C7), 148.3 (C4), 131.6 (C2’,C6’), 121.2, 115.6 (C3’,C5’), 113.0, 110.0 (C3), 99.7 (C8), 95.4 (C6), 56.5 (OCH_3), 55.4 (OCH_3). **MS** (ESI) *m/z*: $[M]^+$ Calcd for $C_{17}H_{15}O_4^+$ 283.0965; Found 283.0958.

(**b**) Orange solid (0.048g, 56 % yield). **^{1}H NMR** (600 MHz, MeOD) *δ* 9.01 (d, *J* = 8.8 Hz, 1H, H4), 8.26 (d, *J* = 9.1 Hz, 2H, H2’,H6’), 8.04 (d, *J* = 8.8 Hz, 1H, H4), 7.10 (d, *J* = 2.2 Hz, 1H, H8), 7.06 (d, *J* = 9.1 Hz, 2H, H3’,H5’), 6.53 (d, *J* = 2.2 Hz, 1H, H6) 3.89 (s, 3H, C4’-OCH_3), 3.79 (s, 3H,C7-OCH_3). **^{13}C{^{1}H} NMR** (151 MHz, MeOD) δ 172.0 (C8a), 171.5 (C2), 167.0 (C4’), 159.0 (C7), 158.5 (C5), 149.2 (C4), 131.8 (C2’,C6’), 121.2, 115.6, (C3’,C5’), 113.4, 110.6 (C3), 101.6 (C6), 92.7 (C8), 56.6 (OCH_3), 55.4 (OCH_3). **MS** (ESI) *m/z*: $[M]^+$ Calcd for $C_{17}H_{15}O_4^+$ 283.0965; Found 283.0958.

(**c**) Orange solid (0.040g, 45 % yield). **^{1}H NMR** (400 MHz, MeOD) *δ* 8.96 (dd, *J* = 8.9, 0.8 Hz, 1H, H4), 8.21 (d, *J* = 9.0 Hz, 2H, H2’, H6’), 8.04 (d, *J* = 8.9 Hz, 1H, H3), 7.18 (dd, *J* = 2.1, 0.8 Hz, 1H, H6), 6.90 (d, *J* = 9.0 Hz, 2H, H3’,H5’), 6.71 (d, *J* = 2.1 Hz, 1H, H8), 3.93 (s, 3H, OCH_3), 3.91 (s, 3H, OCH_3). **^{13}C{^{1}H} NMR** (101 MHz, MeOD) δ 172.4 (C8a), 171.1 (C2), 166.6 (C4’), 158.9 (C5), 158.8 (C7), 148.1 (C4), 132.5 (C2’,C6’), 119.8, 117.2 (C3’,C5’), 113.0, 111.1 (C3), 99.5 (C8), 93.2 (C6), 56.7 (OCH_3), 56.6 (OCH_3). **MS** (ESI) *m/z*: $[M]^+$ Calcd for $C_{17}H_{15}O_4^+$ 283.0965; Found 283.0958.

4. Spectroscopic measurements

The Varian-Cary 100 Bio and 5000 spectrophotometers (Palo Alto, CA, USA) were used to record the UV-Vis spectra. The stopped flow experiments were performed on a SX20 Applied Photophysics (Surrey, UK) spectrometer equipped with a PDA 1/UV photodiode array detector

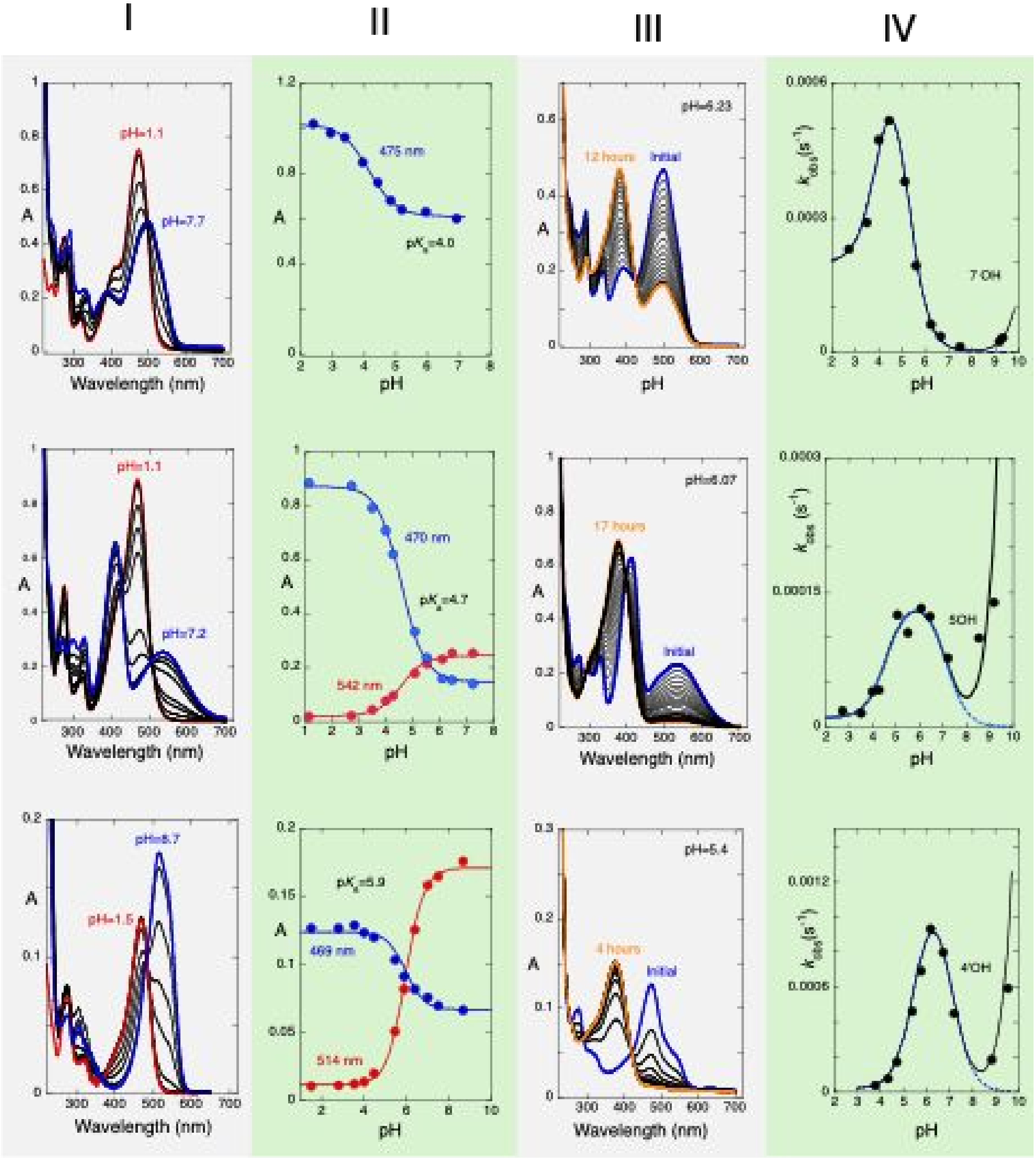

Figure S3. **Column I:** Spectral variations of the absorption spectra versus pH taken 10 ms after a direct pH jump monitored by stopped flow; **Column II**: Determination of the acidity constant; **Column III**: Spectral variations after a direct pH jump for a selected pH; **Column IV**: Bell-shaped curve of the rate constant toward the equilibrium

versus pH. First row 7-OH **a**, second row 5-OH **b**; third row 4'-OH **c**. See Scheme 6 for the meaning of columns I, II, III, IV.

5. NMR Spectra

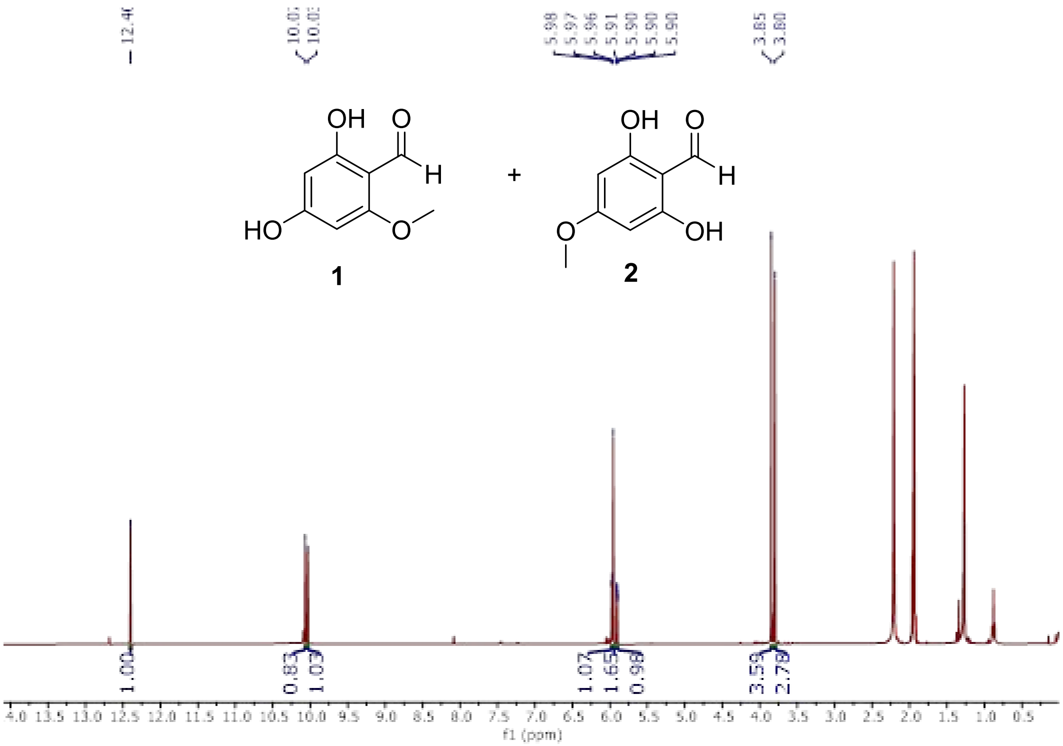

Figure S4. ^{1}H NMR spectrum (CD_3CN) of a mixture of 2,6-dihydroxy-4-methoxybenzaldehyde (**1**) and 2,4-dihydroxy-6-methoxybenzaldehyde (**2**) performed in a Bruker Avance 400 MHz spectrometer.

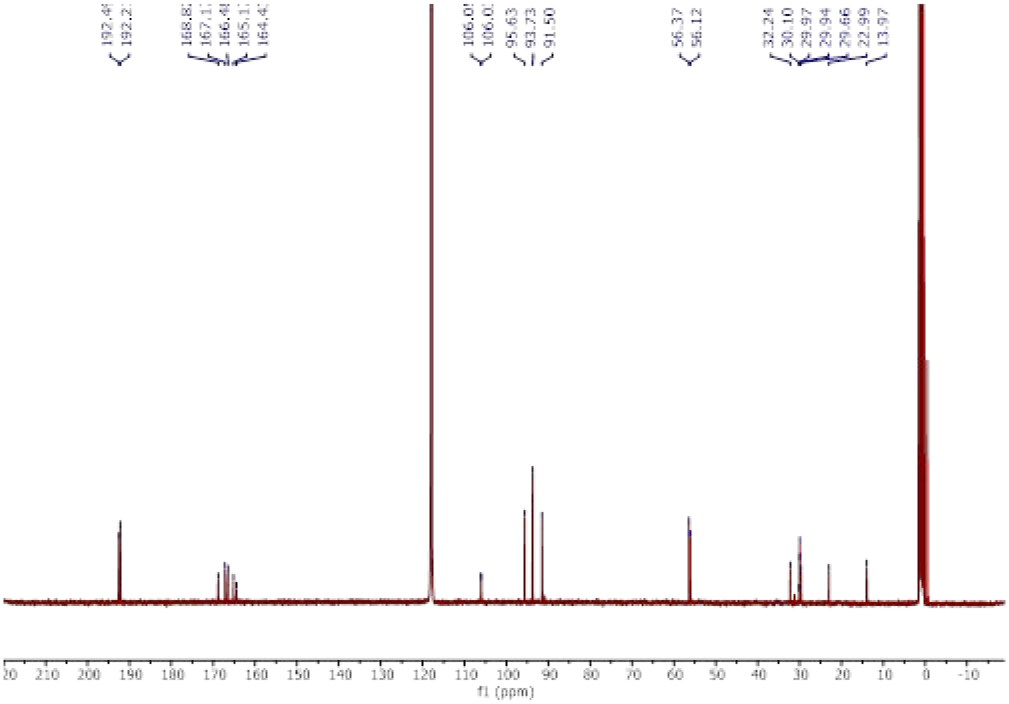

Figure S5. $^{13}C\{^{1}H\}$ NMR spectrum (CD_3CN) of a mixture of 2,6-dihydroxy-4-methoxybenzaldehyde (**1**) and 2,4-dihydroxy-6-methoxybenzaldehyde (**2**) performed in a Bruker Avance 400 MHz spectrometer.

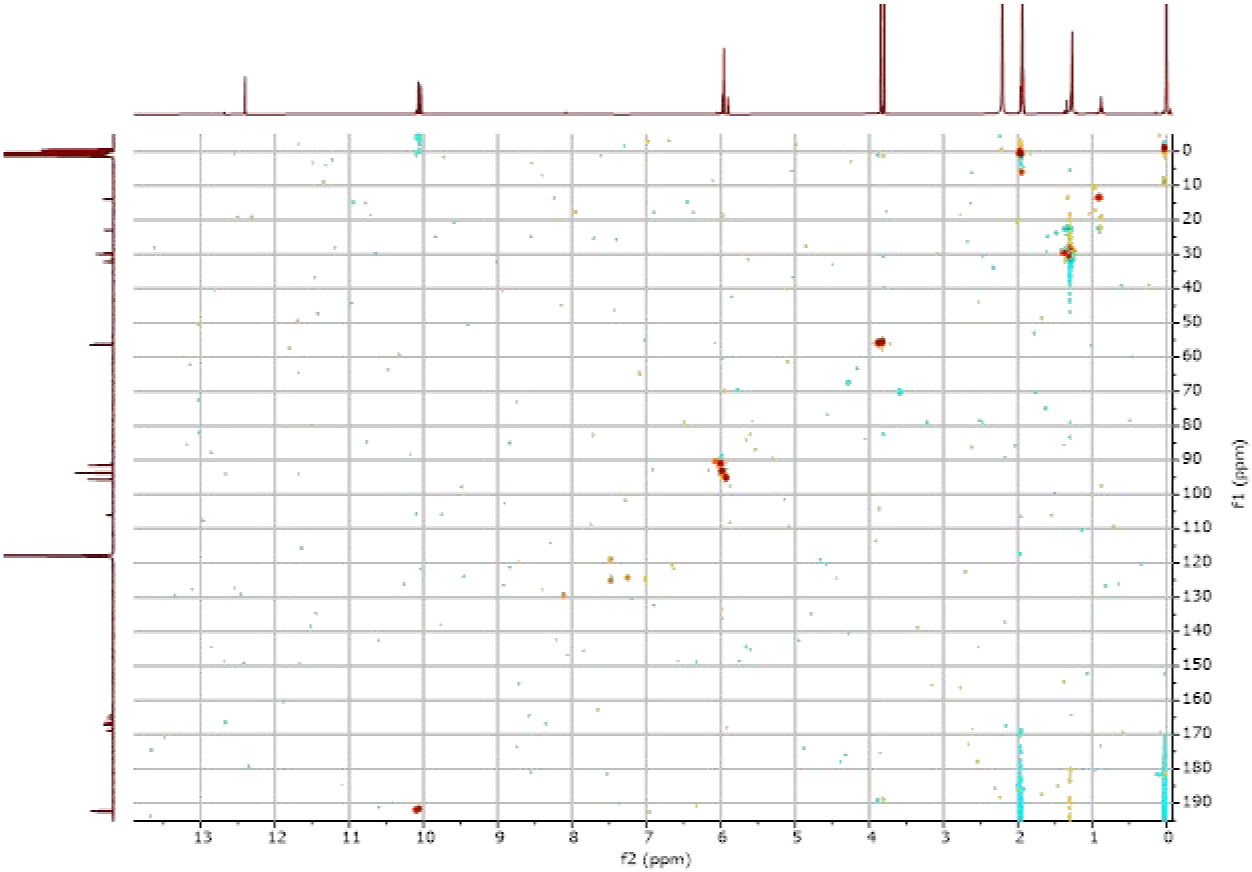

Figure S6. HSQC spectrum (CD_3CN) of a mixture of 2,6-dihydroxy-4-methoxybenzaldehyde (**1**) and 2,4-dihydroxy-6-methoxybenzaldehyde (**2**) performed in a Bruker Avance 400 MHz spectrometer

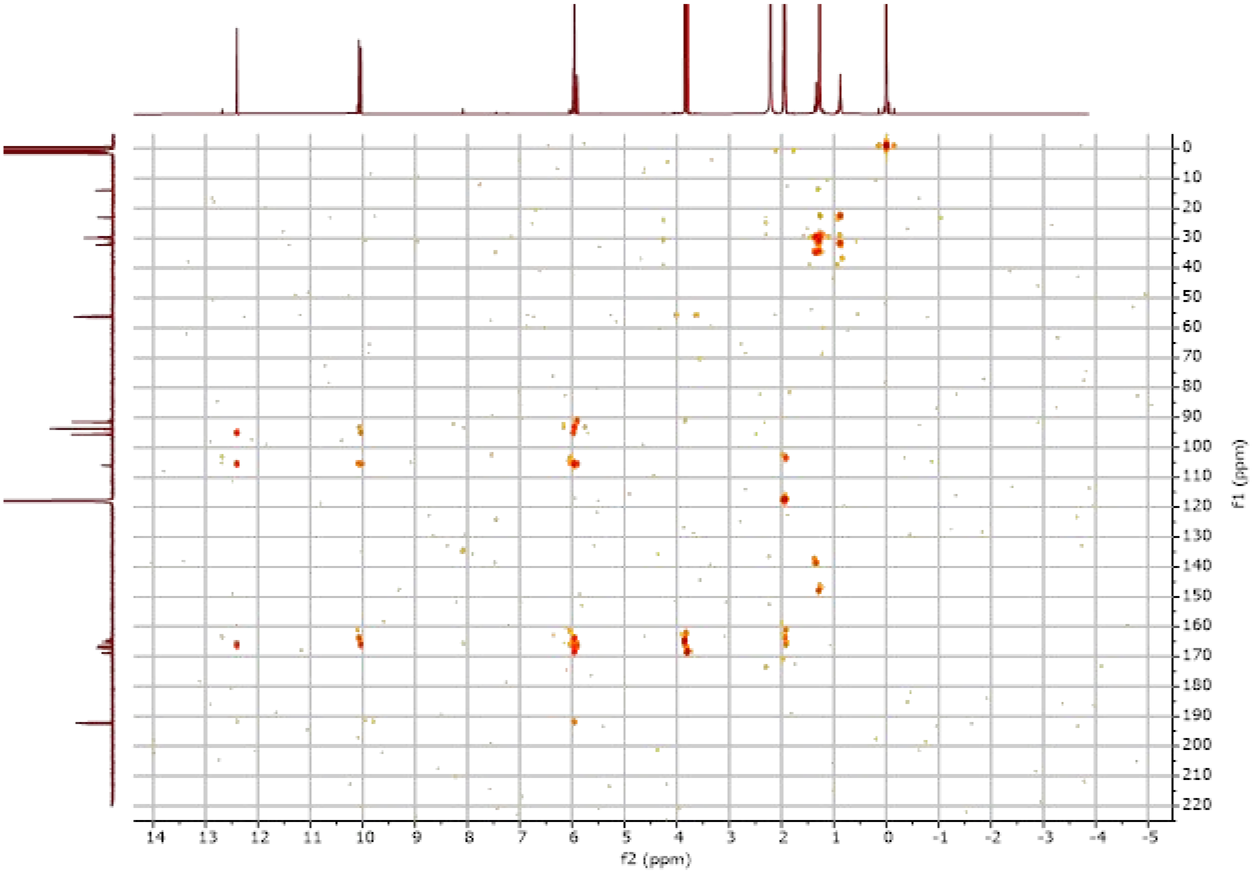

Figure S7. HMBC spectrum (CD_3CN) of a mixture of 2,6-dihydroxy-4-methoxybenzaldehyde (**1**) and 2,4-dihydroxy-6-methoxybenzaldehyde (**2**) performed in a Bruker Avance 400 MHz spectrometer

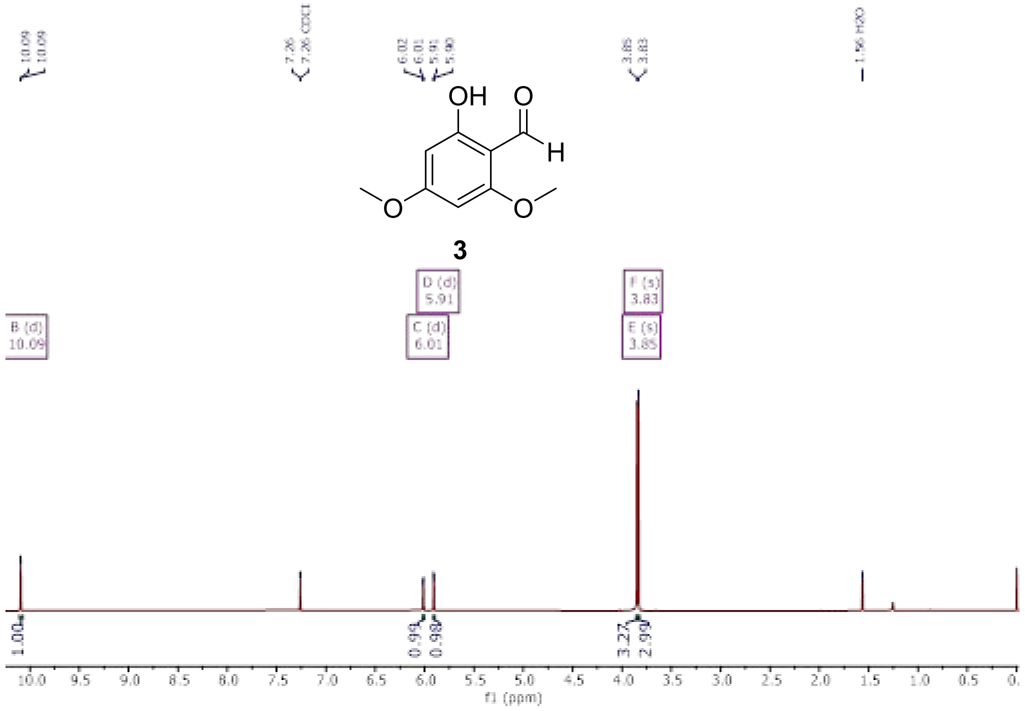

Figure S8. ^{1}H NMR spectrum ($CDCl_3$) of 2-hydroxy-4,6-dimethoxybenzaldehyde (**3**) performed in a Bruker Avance 400 MHz spectrometer.

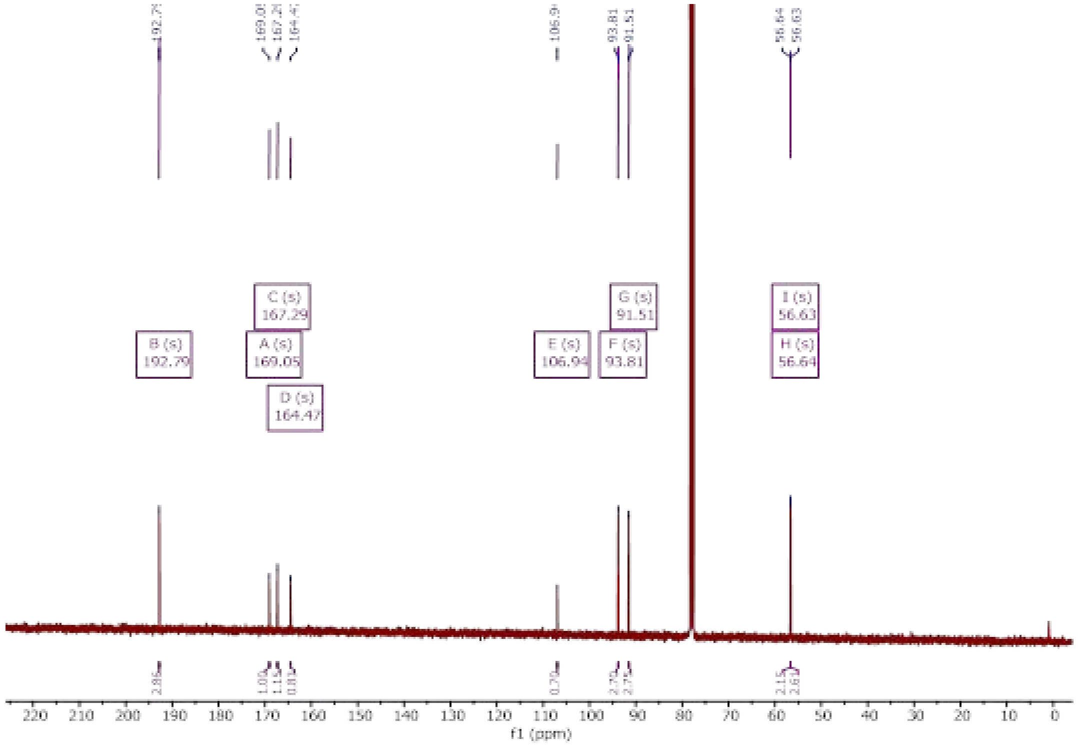

Figure S9. $^{13}C\{^{1}H\}$ NMR spectrum ($CDCl_3$) of 2-hydroxy-4,6-dimethoxybenzaldehyde (**3**) performed in a Bruker Avance 400 MHz spectrometer.

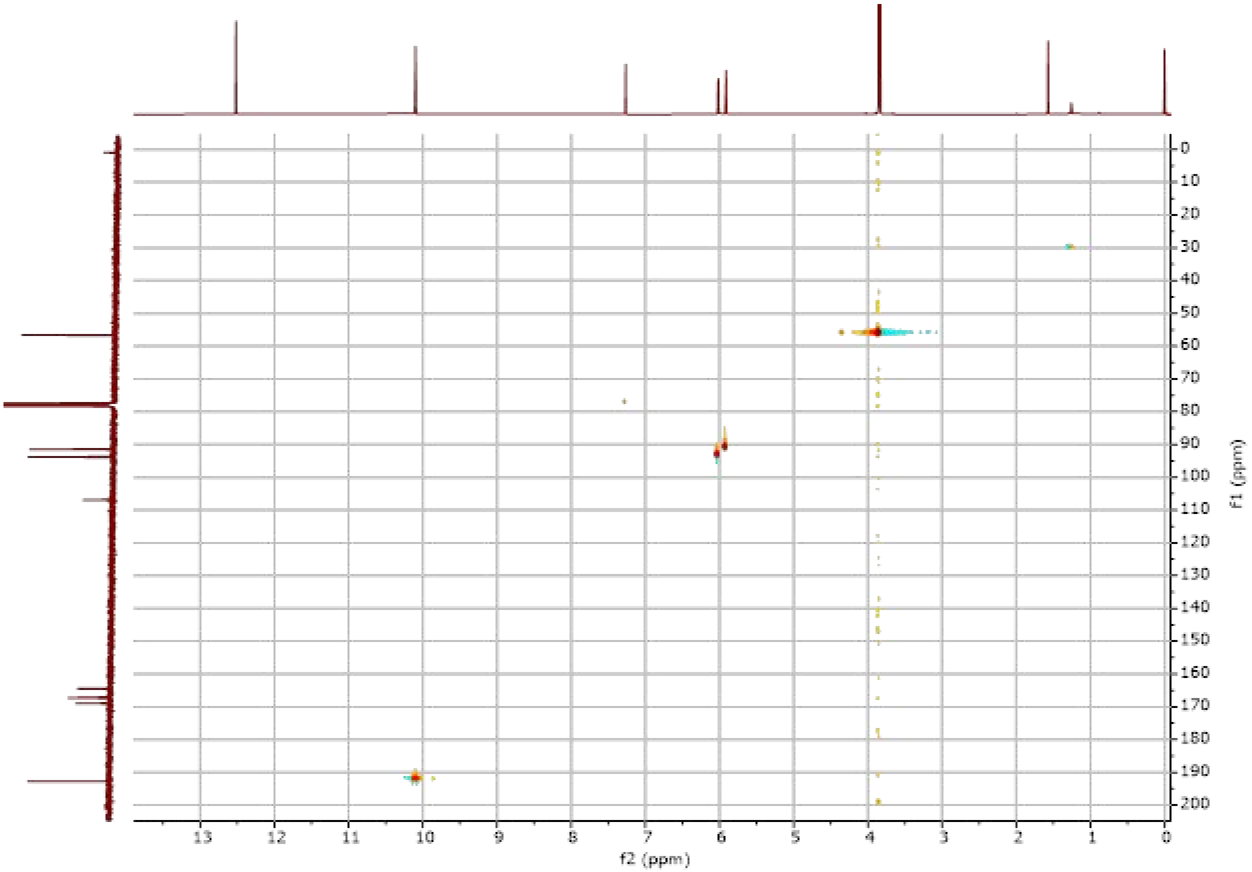

Figure S10. HSQC spectrum ($CDCl_3$) of 2-hydroxy-4,6-dimethoxybenzaldehyde (**3**) performed in a Bruker Avance 400 MHz spectrometer.

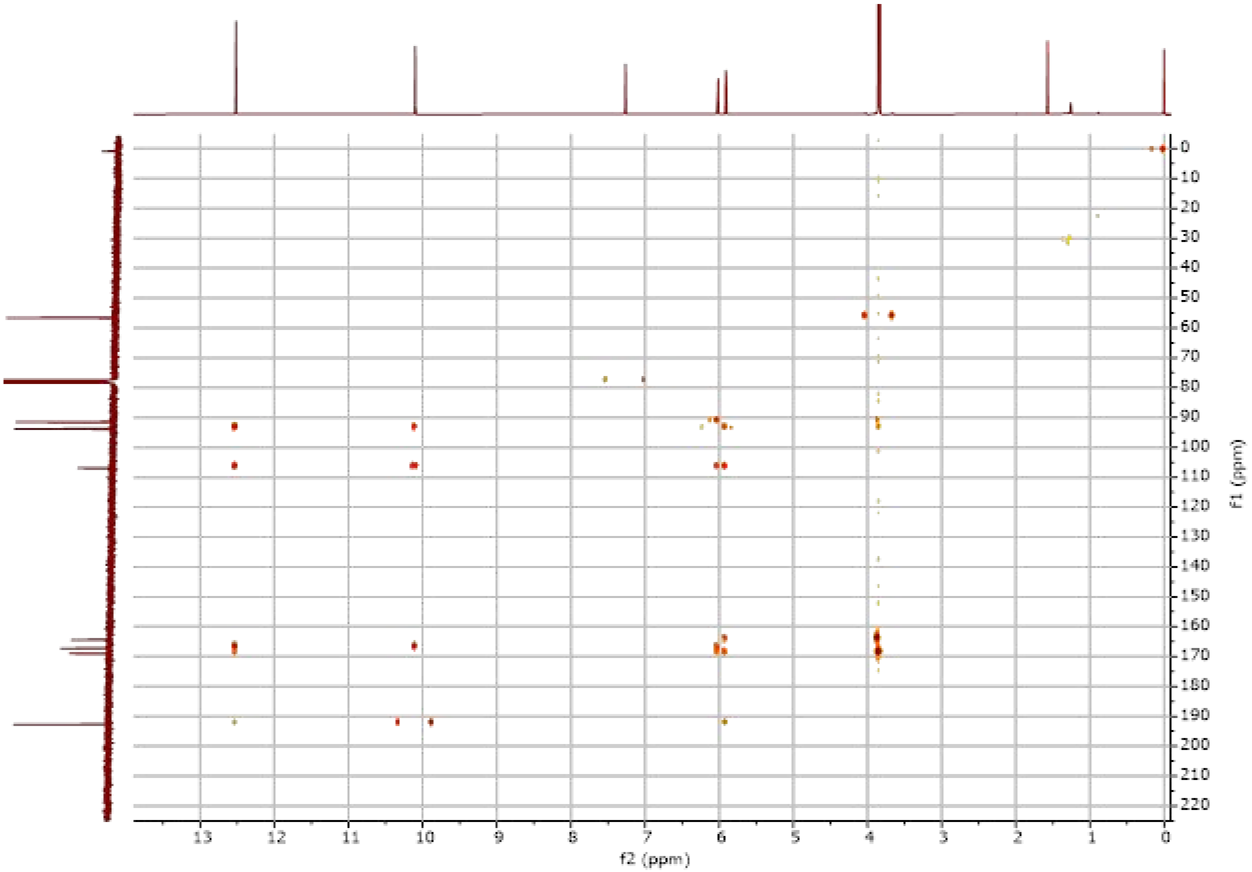

Figure S11. HMBC spectrum ($CDCl_3$) of 2-hydroxy-4,6-dimethoxybenzaldehyde (**3**) performed in a Bruker Avance 400 MHz spectrometer.

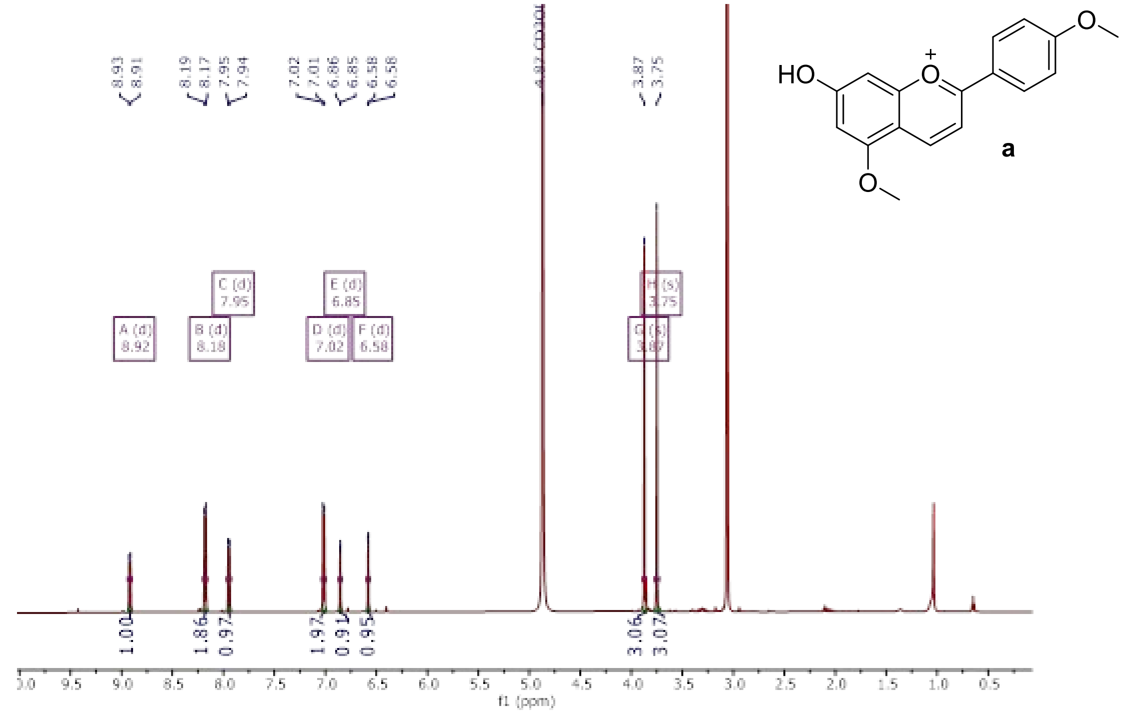

Figure S12. ^{1}H NMR spectrum (MeOD) of 7-hydroxy-4',5-dimethoxyflavylium (**a**) performed in a Bruker Avance 600 MHz spectrometer.

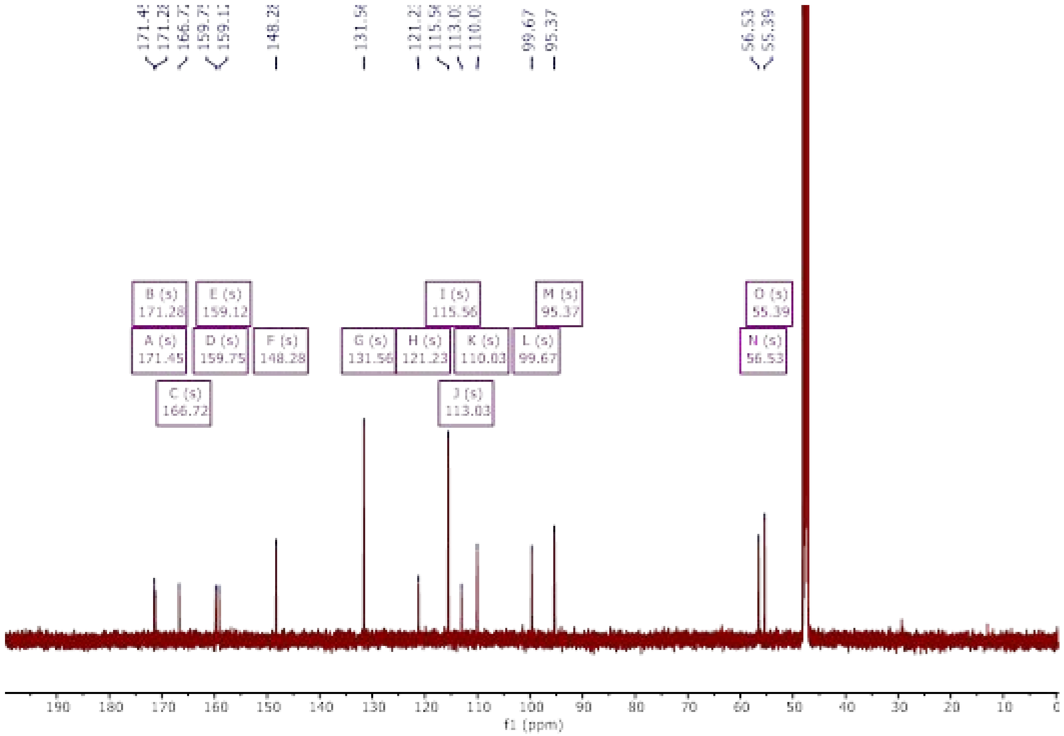

Figure S13. $^{13}C\{^{1}H\}$ NMR spectrum (MeOD) of 7-hydroxy-4',5-dimethoxyflavylium (**a**) performed in a Bruker Avance 600 MHz spectrometer.

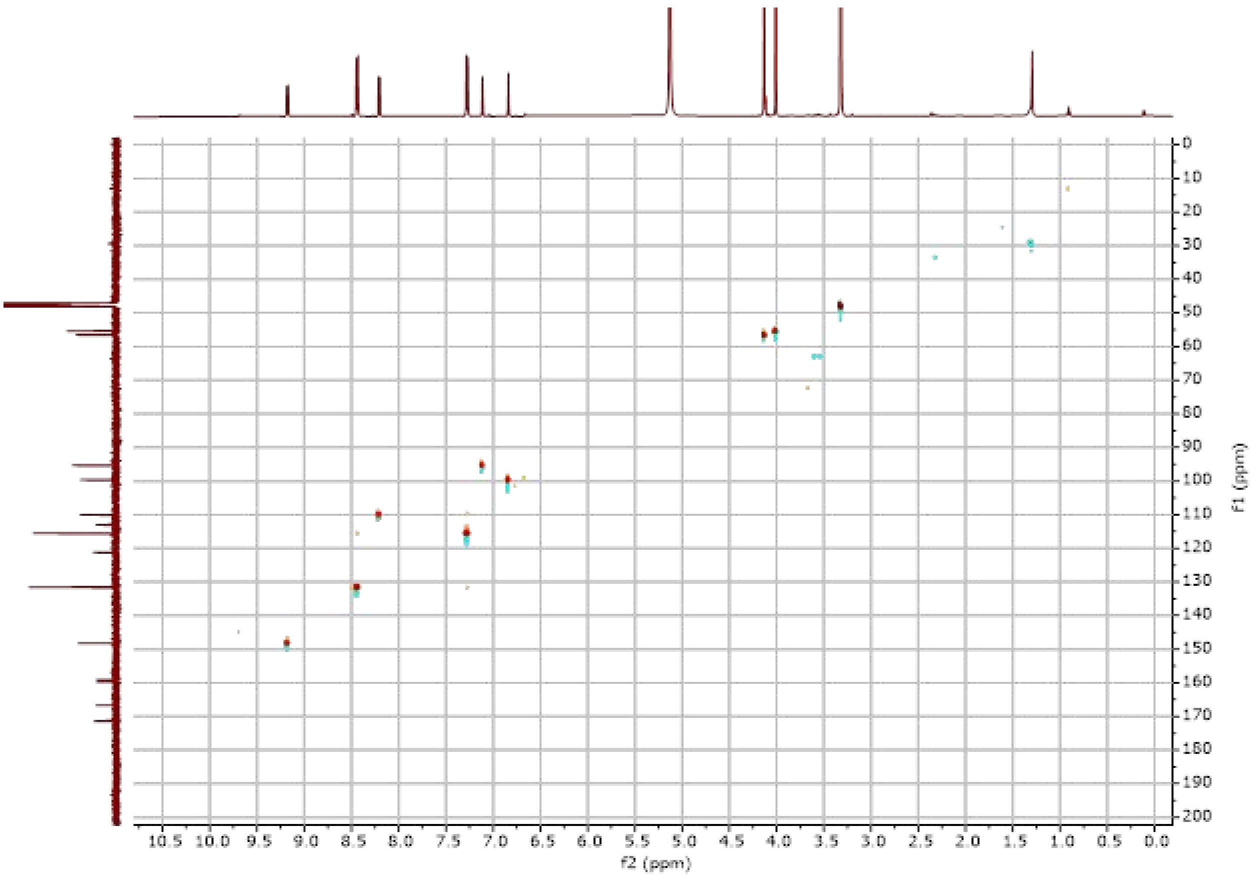

Figure S14. HSQC spectrum *(*MeOD*)* of 7-hydroxy-4',5-dimethoxyflavylium (**a**) performed in a Bruker Avance 600 MHz spectrometer.

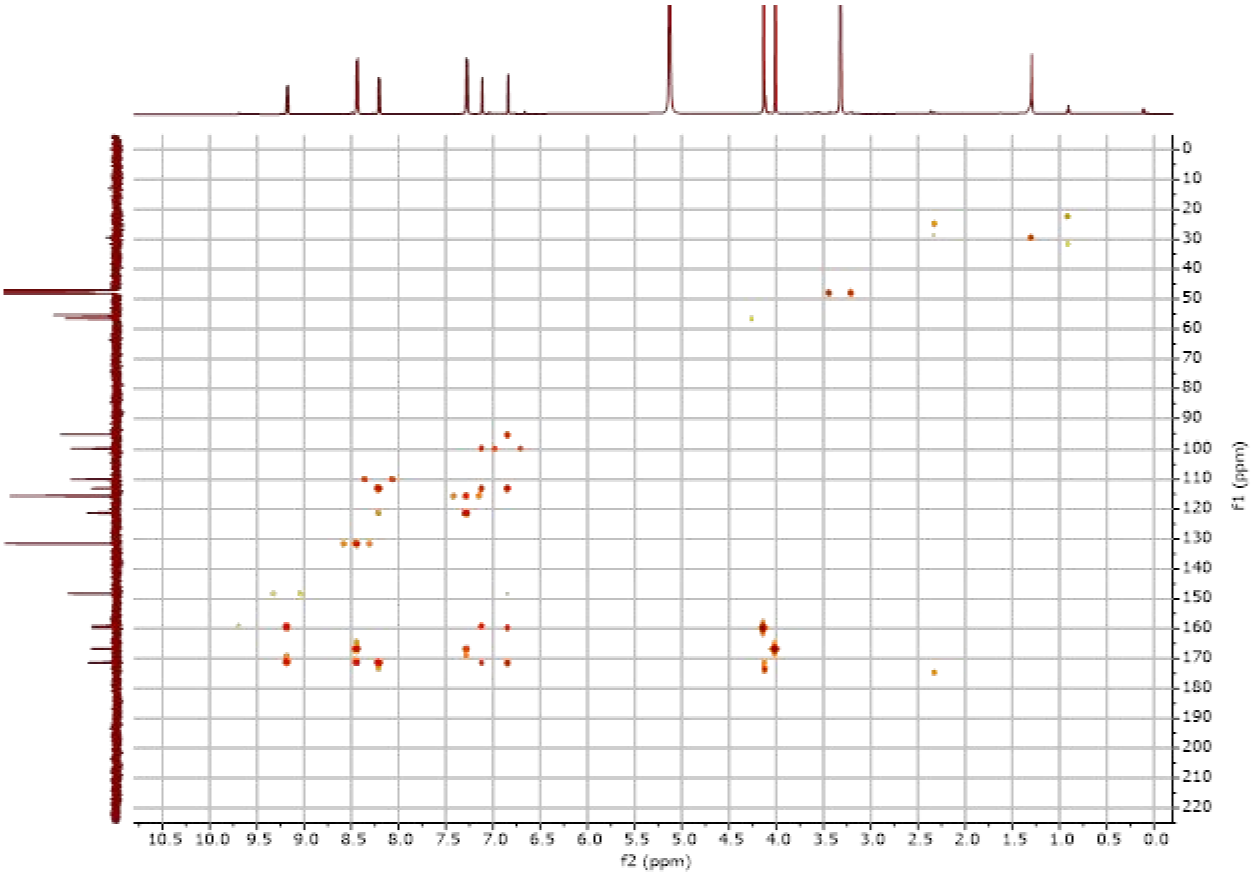

Figure S15. HBMC spectrum (MeOD) of 7-hydroxy-4',5-dimethoxyflavylium (**a**) performed in a Bruker Avance 600 MHz spectrometer.

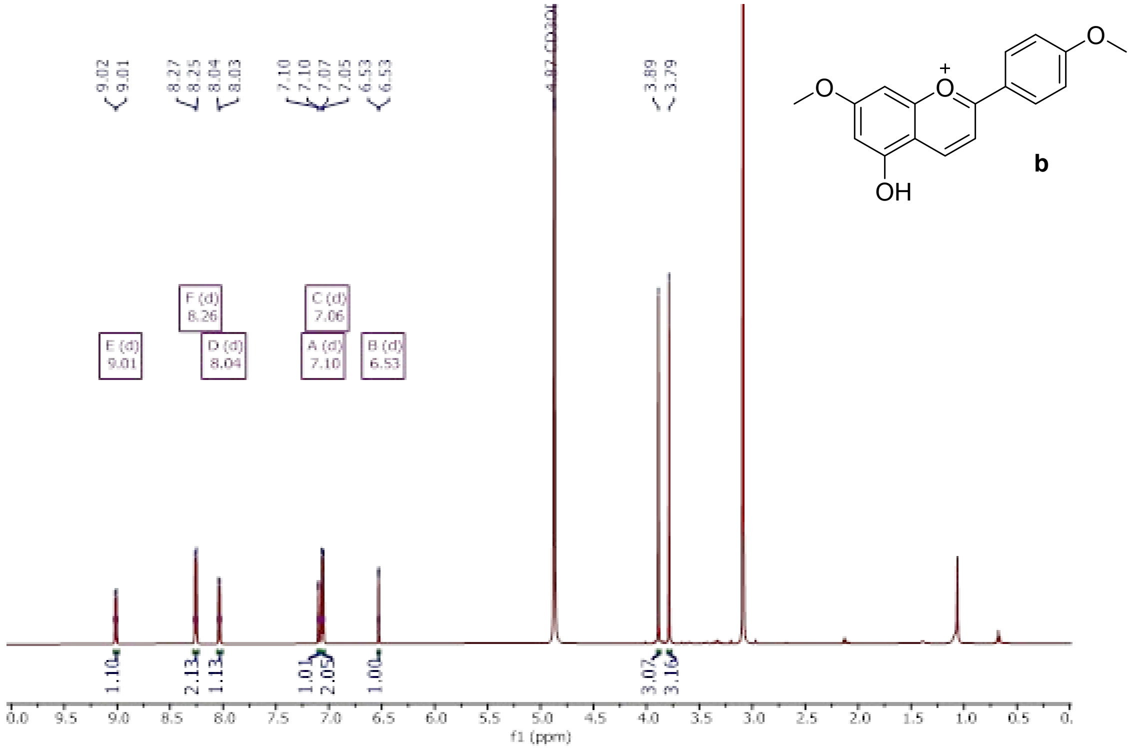

Figure S16. ^{1}H NMR spectrum (MeOD) of 5-hydroxy-4',7-dimethoxyflavylium (**b**) performed in a Bruker Avance 600 MHz spectrometer.

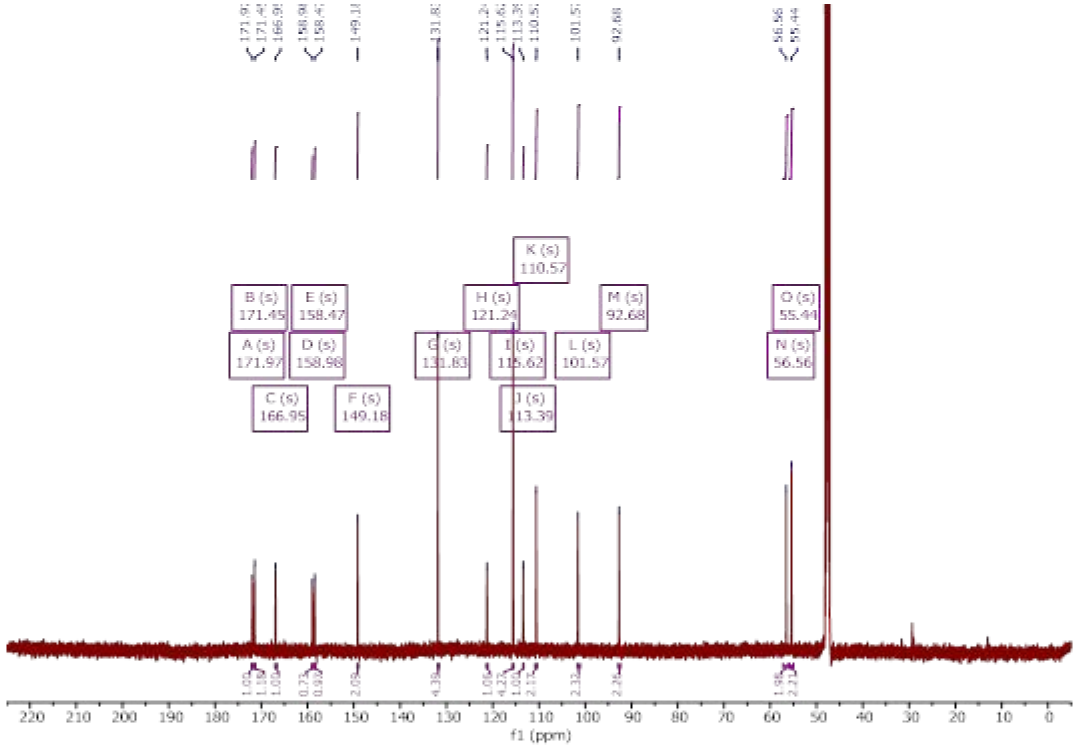

Figure S17. $^{13}C\{^{1}H\}$ NMR spectrum (MeOD) of 5-hydroxy-4',7-dimethoxyflavylium (**b**) performed in a Bruker Avance 600 MHz. spectrometer.

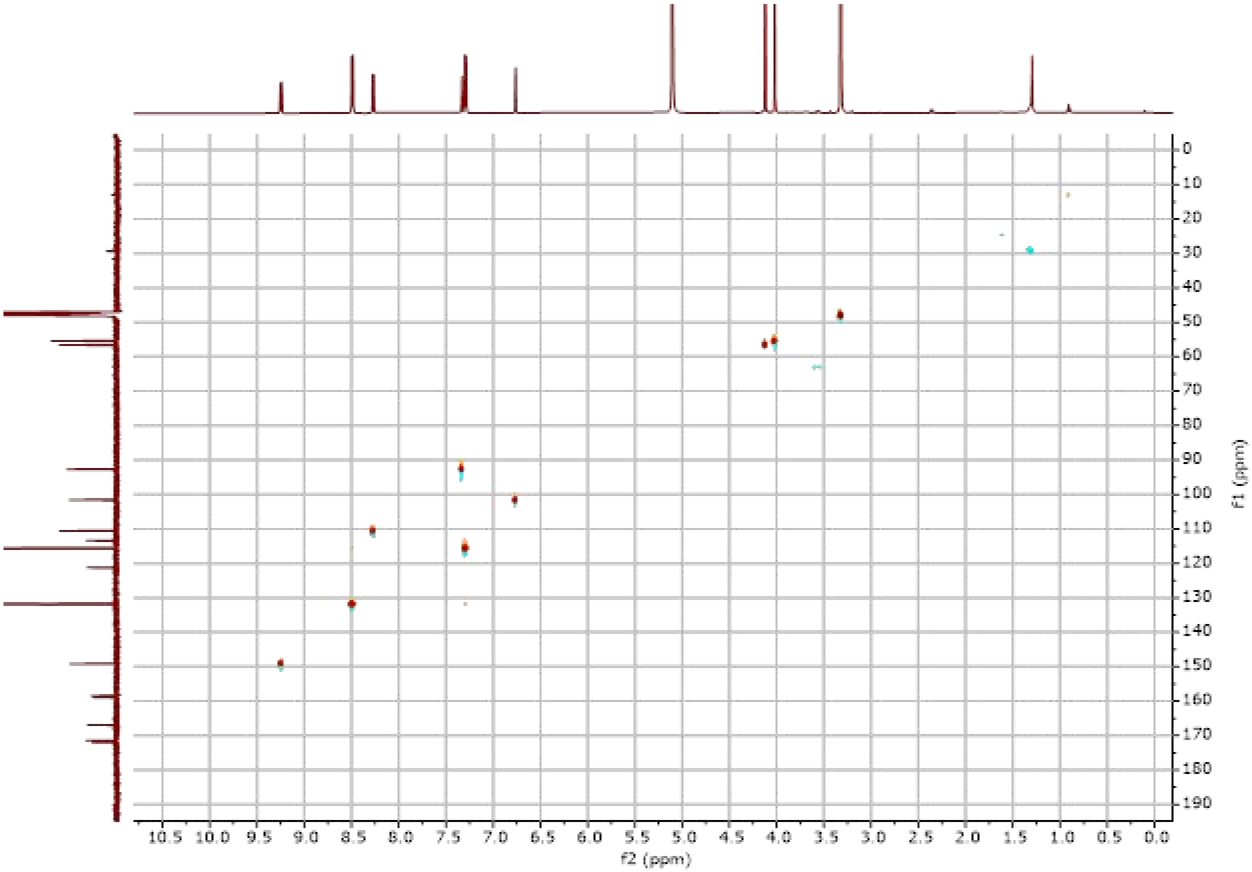

Figure S18. HSQC spectrum (MeOD) of 5-hydroxy-4',7-dimethoxyflavylium (**b**) performed in a Bruker Avance 600 MHz spectrometer.

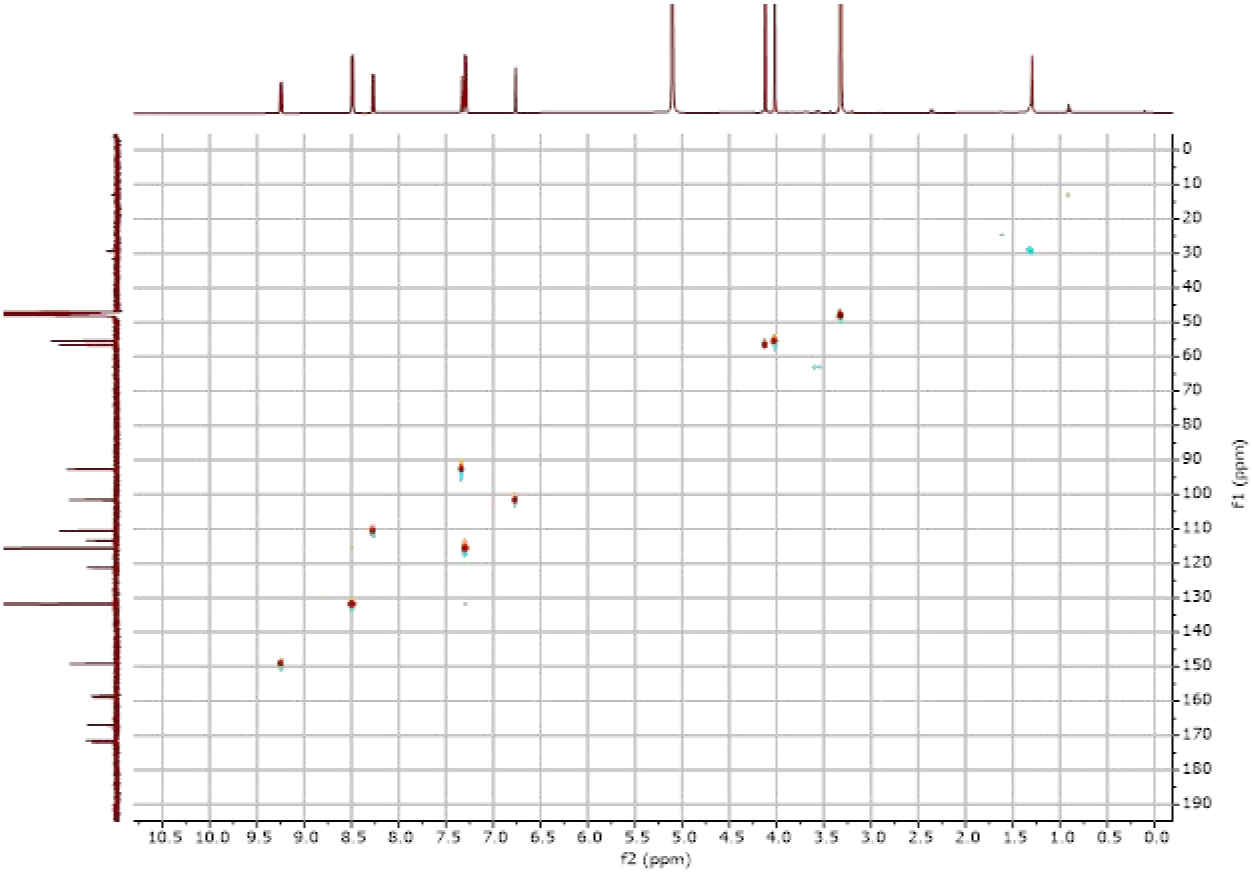

Figure S19. HBMC spectrum (MeOD) of 5-hydroxy-4’,7-dimethoxyflavylium (**b**) performed in a Bruker Avance 600 MHz spectrometer.

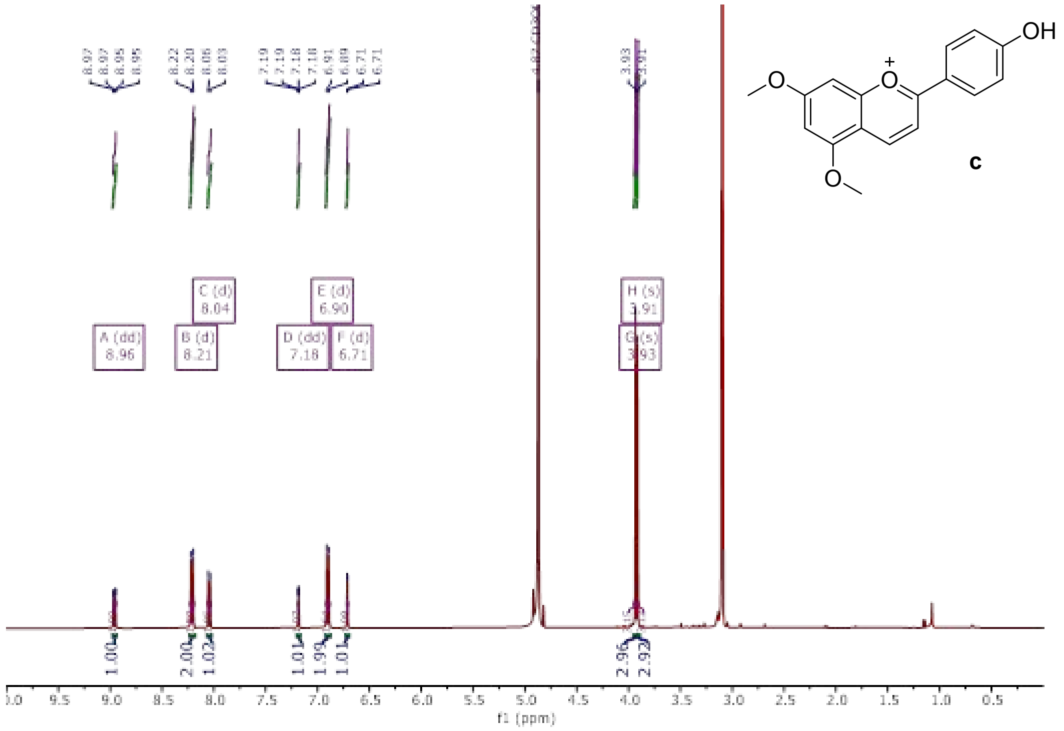

Figure S20. ^{1}H NMR spectrum (MeOD) of 4’-hydroxy-5,7-dimethoxyflavylium (**c**) performed in a Bruker Avance 400 MHz spectrometer.

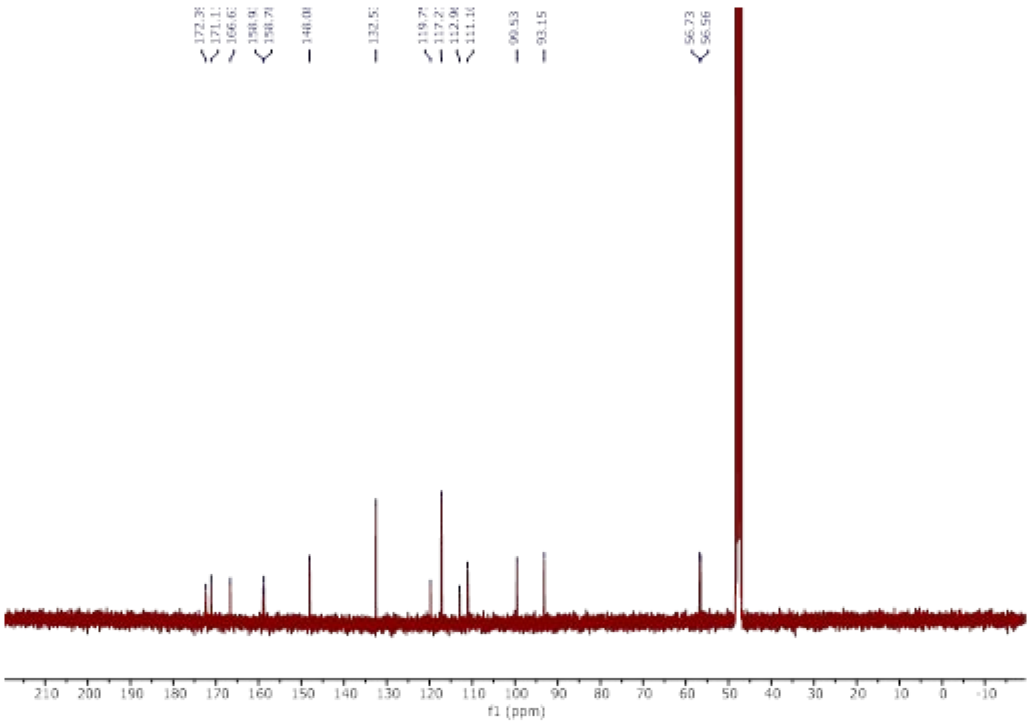

Figure S21. $^{13}C\{^{1}H\}$ NMR spectrum (MeOD) of 4’-hydroxy-5,7-dimethoxyflavylium (**c**) performed in a Bruker Avance 400 MHz spectrometer.

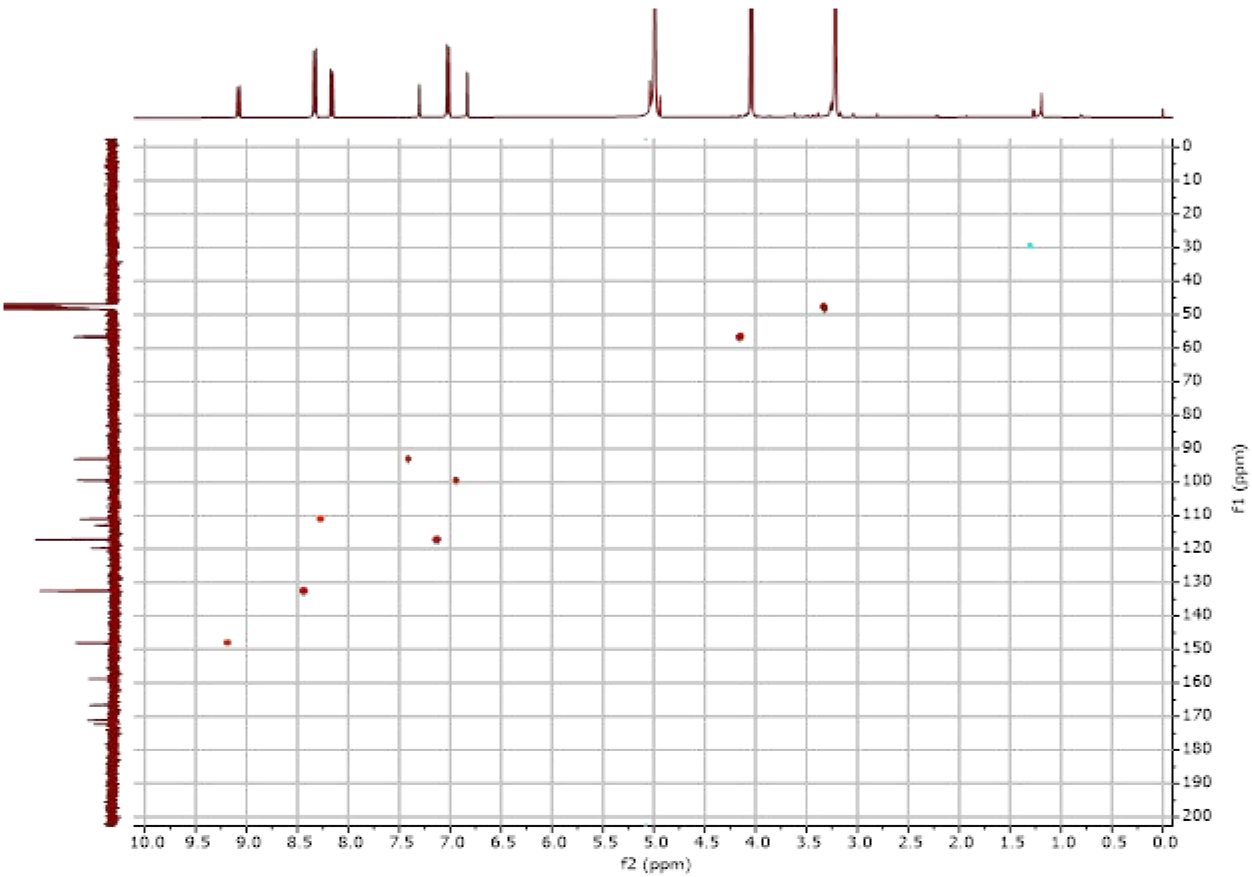

Figure S22. HSQC spectrum (MeOD) of 4'-hydroxy-5,7-dimethoxyflavylium (**c**) performed in a Bruker Avance 400 MHz spectrometer

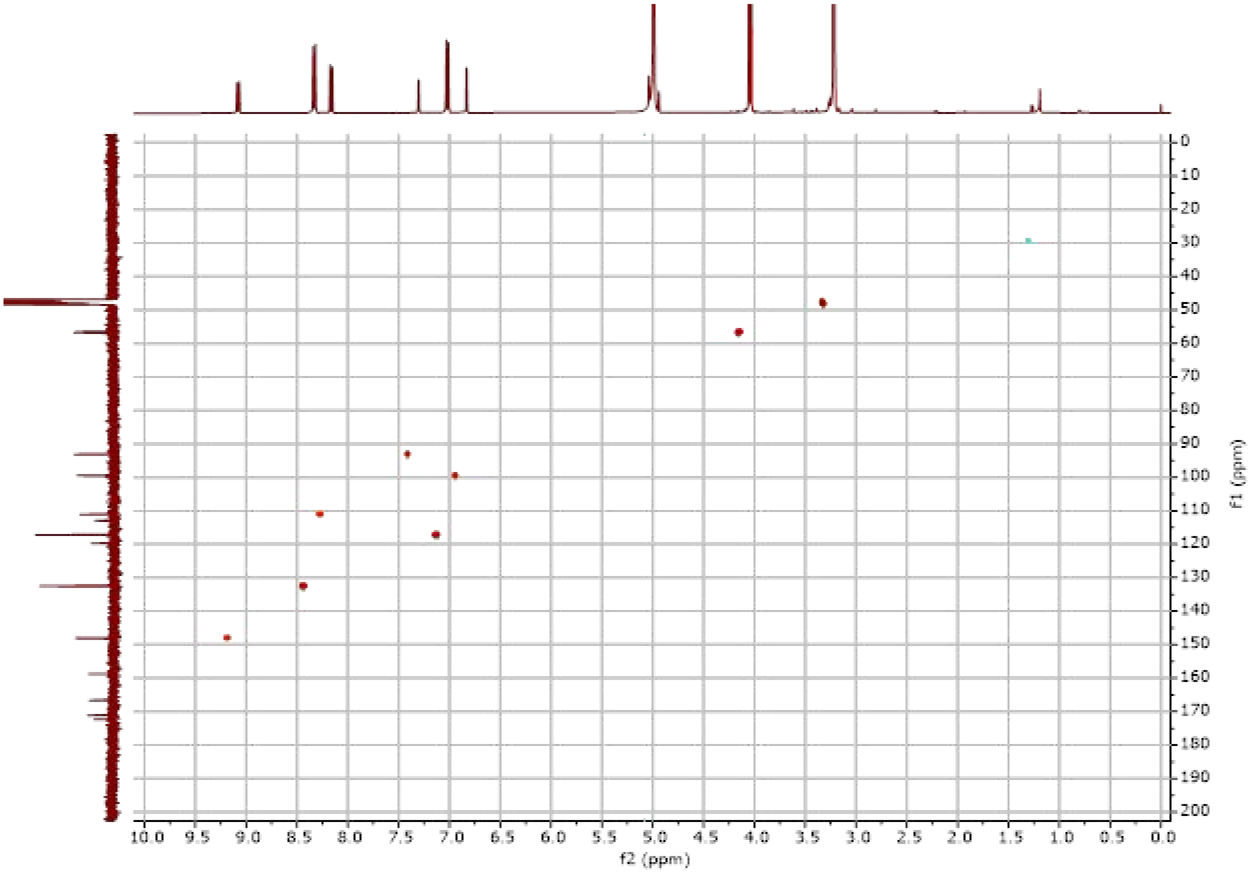

Figure S23. HBMC spectrum (MeOD) of 4’-hydroxy-5,7-dimethoxyflavylium (**c**) performed in a Bruker Avance 400 MHz spectrometer

6. Computational Studies

DFT calculations have been carried out with the program Gaussian 16 using the B3LYP functional and the 6-31++G** basis set. Time–Dependent DFT (TDDFT) calculations were performed to simulate the absorption spectra. All calculations, that is, geometry optimization and TDDFT were performed considering the solvent effect with the universal solvation model based on density (SMD). The binary solvent mixture of H_2O:EtOH was defined by dielectric constant $\varepsilon = 72.42$ and the refractive index $n = 1.3392$ ($\varepsilon_\infty = n^2$) for 20% Ethanol volume at 293.15 K.[9]. In all calculations explicit H_2O molecules, hydrogen bonded to the OH groups were introduced. Molecular structures were drawn using Chemcraft.

6.1. TDDFT Simulated Spectra

In line with the spectrscopic measurements, the very good agreement of the simulated absorption spectra with the experimental UV-Vis supports the reliability of the applied level of theory in our computational studies.

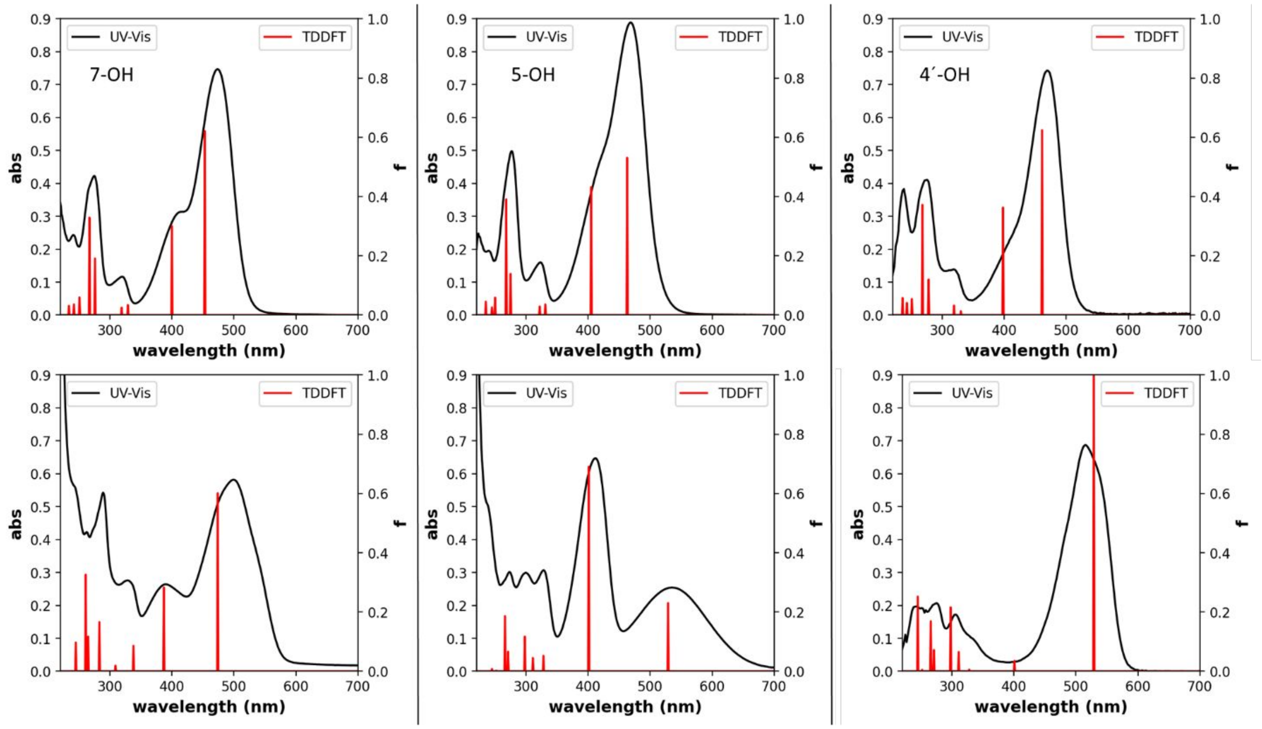

Figure S24. Experimental UV-Vis vs. TDDFT calculated spectra of model molecules. The second row corresponds to the respective quinoidal base.

6.2. ΔpK_a Evaluation

In our adapted method, the O–H distance was increased gradually in a discrete H_2O molecule hydrogen bonded to the carbonyl group of the quinoidal base till a proton of the water is transferred to the base forming the corresponding flavylium cation and OH^- (Fig. S25, Fig. S26).

The advantage of our adapted methodology for evaluation of ΔE (see main text) is that the whole charge of the system is maintained zero, as the solvation free energy of ionic species is not usually straightforward when implicit polarizable continuum solvent models are used for pK_a evaluation.[10] Moreover, our method does not require an arbitrary chosen value of H^+ energy which can introduce a source of error in calculations.[11] We would like to highlight although polarizable continuum solvent models with Bondi atomic radii can improve the calculations in the pK_a studies [10,11,12], the performance of the SMD solvatation with Bondi atomic radii had a negligible impact on ΔpK_a determination in our case. We determined 1.1 (exp 1.9), 0.9 (exp 1.2) and 0.2 (exp 0.7) ΔpK_a leading to a mean absolute error of 0.5 quite acceptable in quantum chemical evaluation of pK_a.[11]

To the observed ΔE values (ΔE = ΔE (in eV) / 0.059 V[13]) the Gompertz function $f(x) = a.exp(-b.exp(-c.x))$ was fitted, where x is the O–H distances and a is an asymptote. The fitted values of a are shown in Fig. S25 and Fig. S26. For the physical meaning of the asymptote see the main text.

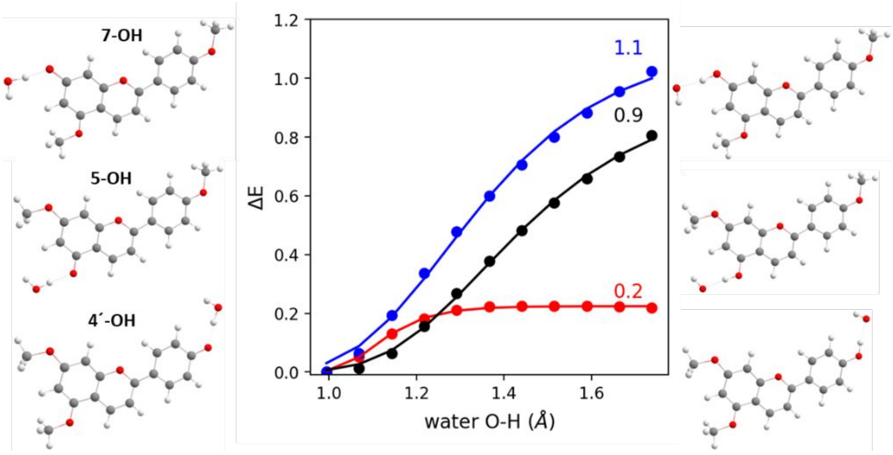

Figure S25. [E(7-OH) – E(4´-OH)] blue dots, [E(5-OH) – E(4´-OH)] black dots and [E(7-OH) – E(5-OH)] red dots in model molecules as a function of water O–H distance. The asymptotic value of each sigmoidal fitted curve (solid lines) are shown. The initial and final geometries in the O–H scanning process are shown on the left and right sides respectively.

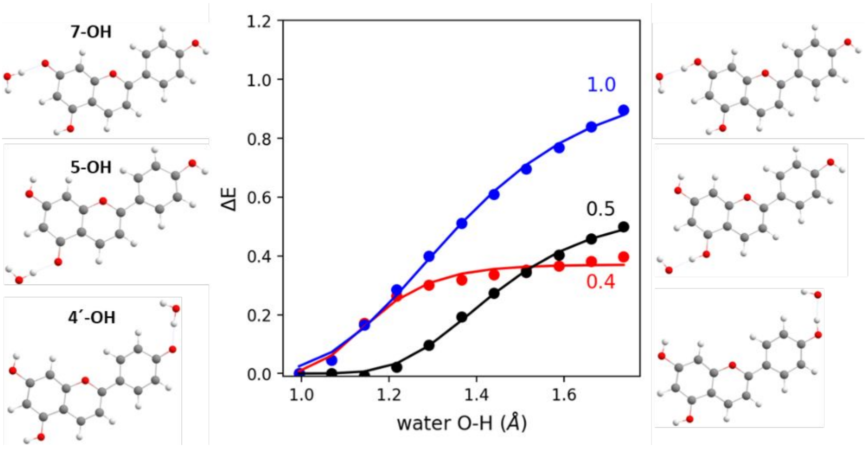

Figure S26. [E(7-OH) – E(4´-OH)] blue dots, [E(5-OH) – E(4´-OH)] black dots and [E(7-OH) – E(5-OH)] red dots in apigeninidin as a function of water O–H distance. The asymptotic value of each sigmoidal fitted curve (solid lines) are shown. The initial and final geometries in the O–H scanning process are shown on the left and right sides, respectively.

6.3. Cartesian coordinates (optimized at the B3LYP (SMD, solvent=Read (eps=72.42, epsinf=1.793))/6-31++G** level), number of imaginary frequencies (NIMAG) and computed total energies of optimized structures.

Synthesized model compound-7OH···H_2O

Energy : -1034.08353099 A.U.

NIMAG = 0

Charge = +1 Multiplicity = 1

Stoichiometry $C_{17}H_{17}O_5$

C	6.856211000	1.365675000	-0.003839000
O	6.304780000	0.035005000	-0.000468000
C	4.957052000	-0.117635000	-0.000377000
C	4.489551000	-1.446417000	0.003740000
C	3.132284000	-1.705964000	0.004524000
C	4.040638000	0.948393000	-0.004002000
C	2.678142000	0.678258000	-0.003544000
C	2.191608000	-0.646564000	0.000845000
C	0.767217000	-0.914583000	0.001967000

O	-0.000522000	0.193931000	-0.007063000
C	-1.367102000	0.160710000	-0.007637000
C	-2.029128000	1.376299000	-0.016658000
C	-3.429997000	1.339345000	-0.017968000
O	-4.173809000	2.460474000	-0.025673000
C	-4.146125000	0.116540000	-0.011572000
C	-3.462457000	-1.084472000	-0.001363000
O	-4.045143000	-2.303558000	0.006838000
C	-5.483968000	-2.384164000	0.005723000
C	-2.026718000	-1.095446000	0.001357000
C	-1.235105000	-2.252959000	0.011483000
C	0.149795000	-2.166050000	0.011969000
H	7.937314000	1.229587000	-0.003167000
H	6.549226000	1.912823000	0.892436000
H	6.549767000	1.908162000	-0.903121000
H	5.208979000	-2.258580000	0.006304000
H	2.805811000	-2.739172000	0.007767000
H	4.378601000	1.976922000	-0.007038000
H	1.983215000	1.509604000	-0.006214000
H	-1.482461000	2.311436000	-0.022588000
H	-3.617760000	3.291505000	-0.014875000
H	-5.227522000	0.159744000	-0.013408000
H	-5.711600000	-3.449515000	0.013314000
H	-5.893181000	-1.920171000	-0.896369000
H	-5.895235000	-1.906907000	0.899956000
H	-1.709122000	-3.227887000	0.018807000
H	0.750432000	-3.064349000	0.019580000
O	-2.895809000	4.797784000	0.021253000
H	-2.425173000	4.910074000	0.861555000
H	-2.208658000	4.861834000	-0.659923000

Synthesized model compound-5OH···H_2O

Energy : -1034.08250369 A.U.

NIMAG = 0

Charge = +1 Multiplicity = 1

Stoichiometry $C_{17}H_{17}O_5$

C	-7.143967000	1.403316000	0.000943000
O	-6.563909000	0.084540000	0.000243000
C	-5.213757000	-0.039787000	-0.000133000
C	-4.718152000	-1.358579000	-0.000373000
C	-3.355807000	-1.589322000	-0.000619000
C	-4.320152000	1.045643000	-0.000281000
C	-2.952631000	0.804589000	-0.000600000
C	-2.437720000	-0.509869000	-0.000752000
C	-1.008098000	-0.745100000	-0.000658000
O	-0.267075000	0.380193000	-0.001845000
C	1.102740000	0.376148000	-0.001181000
C	1.741876000	1.598345000	-0.002158000
C	3.146808000	1.591788000	-0.000795000
O	3.725767000	2.813801000	-0.001453000
C	5.163812000	2.922576000	0.000809000
C	3.885850000	0.389453000	0.001308000
C	3.218730000	-0.829396000	0.001846000
O	3.855404000	-2.015030000	0.003777000
C	1.788736000	-0.868021000	0.000744000
C	1.021519000	-2.043082000	0.001553000
C	-0.363941000	-1.984964000	0.000813000
H	-8.221839000	1.243740000	0.001403000
H	-6.849511000	1.954694000	-0.896889000
H	-6.848653000	1.954100000	0.898858000
H	-5.420300000	-2.185698000	-0.000306000

H	-3.007858000	-2.615548000	-0.000751000
H	-4.680099000	2.066620000	-0.000078000
H	-2.275426000	1.650349000	-0.000620000
H	1.187136000	2.528523000	-0.003537000
H	5.368095000	3.992723000	0.000177000
H	5.586345000	2.462361000	-0.896636000
H	5.583349000	2.464000000	0.900496000
H	4.968221000	0.398258000	0.002684000
H	4.850316000	-1.915066000	0.002367000
H	1.517318000	-3.007347000	0.002901000
H	-0.946958000	-2.894724000	0.001591000
O	6.513720000	-2.011983000	0.009777000
H	6.873916000	-1.643968000	-0.811832000
H	6.869620000	-1.447622000	0.713372000

Synthesized model compound-4´OH···H_2O

Energy : -1034.08255432 A.U.

NIMAG = 0

Charge = +1 Multiplicity = 1

Stoichiometry $C_{17}H_{17}O_5$

O	-6.147482000	-1.162409000	-0.001673000
C	-4.867912000	-0.728648000	-0.001609000
C	-4.532952000	0.639129000	-0.013846000
C	-3.202743000	1.024810000	-0.012665000
C	-3.847021000	-1.698396000	0.010520000
C	-2.520229000	-1.307377000	0.010383000
C	-2.164314000	0.063608000	-0.000719000
C	-0.775668000	0.467471000	-0.000272000
O	0.095107000	-0.560736000	-0.000972000
C	1.453599000	-0.393303000	-0.000422000
C	2.231437000	-1.534334000	-0.001957000

C	3.622990000	-1.364359000	-0.001682000
O	4.343948000	-2.508073000	-0.003903000
C	5.785108000	-2.445678000	-0.004507000
C	4.219213000	-0.082016000	0.000862000
C	3.413334000	1.047458000	0.002529000
O	3.879696000	2.314633000	0.005081000
C	5.304137000	2.532970000	0.006733000
C	1.987382000	0.920960000	0.001540000
C	1.085078000	1.998218000	0.002196000
C	-0.282105000	1.776087000	0.001042000
H	-6.802615000	-0.410237000	-0.010776000
H	-5.319338000	1.386888000	-0.023977000
H	-2.977345000	2.084726000	-0.022432000
H	-4.115622000	-2.749624000	0.019810000
H	-1.746873000	-2.066263000	0.019840000
H	1.787980000	-2.522426000	-0.003753000
H	6.114420000	-3.484079000	-0.006887000
H	6.148897000	-1.941358000	0.895157000
H	6.147917000	-1.937583000	-0.902447000
H	5.294716000	0.015727000	0.001135000
H	5.428042000	3.615285000	0.008860000
H	5.758304000	2.105917000	-0.891941000
H	5.756690000	2.102629000	0.904662000
H	1.462158000	3.014622000	0.003857000
H	-0.968245000	2.611071000	0.002194000
O	-8.064112000	0.714350000	-0.076063000
H	-8.593435000	0.684815000	0.735725000
H	-7.688900000	1.608173000	-0.097548000

Corresponding neutral quinoidal base (model compound-7OH···H_2O)

Energy : -1033.62682011 A.U.

NIMAG = 0

Charge = 0 Multiplicity = 1

Stoichiometry $C_{17}H_{16}O_5$

C	7.067645000	1.625256000	-0.000084000
O	6.542000000	0.285871000	-0.000243000
C	5.191166000	0.113530000	0.000051000
C	4.741803000	-1.220171000	-0.000183000
C	3.386329000	-1.498740000	-0.000097000
C	4.260265000	1.163668000	0.000513000
C	2.898889000	0.873033000	0.000608000
C	2.430662000	-0.455583000	0.000260000
C	1.002345000	-0.745901000	0.000142000
O	0.225565000	0.365218000	0.000176000
C	-1.148102000	0.323097000	-0.000111000
C	-1.822068000	1.517068000	-0.000354000
C	-3.253869000	1.505860000	-0.000569000
O	-3.924345000	2.601798000	-0.001046000
C	-3.933132000	0.226733000	-0.000447000
C	-3.234864000	-0.955303000	-0.000111000
O	-3.796622000	-2.192603000	0.000111000
C	-5.231354000	-2.293831000	0.000119000
C	-1.791190000	-0.956644000	0.000002000
C	-0.995219000	-2.093045000	-0.000004000
C	0.403609000	-1.992322000	0.000021000
H	8.151540000	1.511370000	-0.000288000
H	6.751503000	2.165824000	0.897388000
H	6.751179000	2.166120000	-0.897266000
H	5.471563000	-2.023341000	-0.000499000
H	3.073807000	-2.536543000	-0.000323000
H	4.581527000	2.197698000	0.000768000
H	2.193249000	1.695259000	0.000936000
H	-1.286831000	2.459831000	-0.000427000
H	-5.016107000	0.242396000	-0.000605000
H	-5.446067000	-3.362307000	0.000398000

H	-5.651164000	-1.829332000	-0.897323000
H	-5.651178000	-1.828864000	0.897313000
H	-1.455266000	-3.074768000	0.000002000
H	1.010448000	-2.886763000	-0.000025000
O	-6.633926000	2.680270000	0.001104000
H	-6.972207000	1.773880000	0.000717000
H	-5.644864000	2.584888000	-0.000106000

Corresponding neutral quinoidal base (model compound-5OH···H_2O)

Energy : -1033.62593287 A.U.

NIMAG = 0

Charge = 0 Multiplicity = 1

Stoichiometry $C_{17}H_{16}O_5$

C	7.114029000	1.420763000	0.000385000
O	6.546479000	0.098216000	-0.000206000
C	5.192017000	-0.033076000	-0.000090000
C	4.702277000	-1.352701000	-0.000524000
C	3.339133000	-1.589766000	-0.000492000
C	4.293632000	1.045515000	0.000364000
C	2.924611000	0.796769000	0.000384000
C	2.416028000	-0.517331000	-0.000040000
C	0.979974000	-0.762127000	-0.000007000
O	0.238851000	0.369584000	-0.000126000
C	-1.136984000	0.363659000	-0.000117000
C	-1.777167000	1.577440000	-0.000302000
C	-3.194771000	1.558056000	-0.000165000
O	-3.761966000	2.794321000	-0.000311000
C	-5.196708000	2.909513000	-0.000018000
C	-3.932115000	0.373580000	0.000049000
C	-3.287897000	-0.892422000	0.000137000
O	-3.933506000	-2.003554000	0.000119000

C	-1.822608000	-0.889982000	0.000153000
C	-1.055679000	-2.047869000	0.000292000
C	0.343207000	-1.992822000	0.000204000
H	8.193719000	1.272462000	0.000410000
H	6.814832000	1.970471000	0.898051000
H	6.814967000	1.971196000	-0.896886000
H	5.407327000	-2.177585000	-0.000901000
H	2.995356000	-2.617687000	-0.000853000
H	4.646882000	2.068993000	0.000688000
H	2.244140000	1.639919000	0.000746000
H	-1.232615000	2.513540000	-0.000444000
H	-5.399332000	3.980396000	-0.000052000
H	-5.622014000	2.451091000	0.897514000
H	-5.622392000	2.450967000	-0.897308000
H	-5.014706000	0.388654000	0.000120000
H	-1.552592000	-3.012495000	0.000502000
H	0.924930000	-2.903729000	0.000285000
O	-6.637338000	-2.086995000	-0.000081000
H	-6.976875000	-1.181099000	0.001613000
H	-5.647659000	-1.990854000	0.000078000

Corresponding neutral quinoidal base (model compound-4´OH···H_2O)

Energy : -1033.62336100 A.U.

NIMAG = 0

Charge = 0 Multiplicity = 1

Stoichiometry $C_{17}H_{16}O_5$

O	6.326772000	-0.128340000	-0.029393000
C	5.050062000	-0.291367000	-0.033018000
C	4.461184000	-1.604936000	-0.038899000
C	3.099466000	-1.784485000	-0.037196000
C	4.136611000	0.822645000	-0.030544000

C	2.776017000	0.635266000	-0.031229000
C	2.202610000	-0.672762000	-0.032398000
C	0.793020000	-0.858076000	-0.024796000
O	0.068662000	0.290333000	-0.038859000
C	-1.303225000	0.314154000	-0.025847000
C	-1.908093000	1.558324000	-0.039275000
C	-3.308289000	1.596687000	-0.022007000
O	-3.854950000	2.839668000	-0.033864000
C	-5.287859000	2.983733000	-0.006878000
C	-4.082716000	0.417746000	0.006601000
C	-3.444306000	-0.818295000	0.020039000
O	-4.095546000	-2.006990000	0.050395000
C	-5.535071000	-2.012607000	0.080693000
C	-2.021468000	-0.902119000	0.002593000
C	-1.277732000	-2.109098000	0.013919000
C	0.096478000	-2.088277000	-0.000938000
H	5.127753000	-2.462707000	-0.043391000
H	2.711955000	-2.797569000	-0.041220000
H	4.551661000	1.826571000	-0.027331000
H	2.122893000	1.500969000	-0.027990000
H	-1.323187000	2.469851000	-0.059508000
H	-5.467539000	4.058480000	-0.016536000
H	-5.703969000	2.546542000	0.905367000
H	-5.740474000	2.524386000	-0.890406000
H	-5.160822000	0.476268000	0.020318000
H	-5.817422000	-3.064575000	0.111480000
H	-5.942001000	-1.544203000	-0.820434000
H	-5.903649000	-1.501325000	0.975073000
H	-1.800809000	-3.058322000	0.034975000
H	0.655013000	-3.013572000	0.009372000
O	7.462873000	2.317473000	0.149017000
H	6.989601000	1.444794000	0.080404000
H	6.758355000	2.978073000	0.206554000

Apigeninidin-7OH···H_2O

Energy : -955.4874158 A.U.

NIMAG = 0

Charge = +1 Multiplicity = 1

Stoichiometry $C_{15}H_{13}O_5$

O	6.239936000	1.198105000	-0.001109000
C	4.981848000	0.675077000	-0.000714000
C	4.750659000	-0.709248000	-0.002438000
C	3.450439000	-1.188741000	-0.001858000
C	3.904120000	1.574845000	0.001553000
C	2.606693000	1.090016000	0.002159000
C	2.349651000	-0.301579000	0.000614000
C	0.990473000	-0.805470000	0.001539000
O	0.048688000	0.157990000	-0.002725000
C	-1.293343000	-0.104326000	-0.002634000
C	-2.150863000	0.981400000	-0.006386000
C	-3.527665000	0.710571000	-0.006531000
O	-4.450451000	1.689281000	-0.009804000
C	-4.019843000	-0.616227000	-0.003610000
C	-3.142114000	-1.680599000	0.000288000
O	-3.544992000	-2.978559000	0.003712000
C	-1.730117000	-1.455313000	0.001160000
C	-0.754790000	-2.464386000	0.006144000
C	0.594301000	-2.144424000	0.006629000
H	5.591944000	-1.396160000	-0.004421000
H	3.298659000	-2.261617000	-0.003721000
H	4.099281000	2.642027000	0.002961000
H	1.782136000	1.792777000	0.004192000
H	-1.769199000	1.995133000	-0.009440000
H	-4.046474000	2.604839000	-0.007132000

H	-5.091802000	-0.785030000	-0.004101000
H	-1.058705000	-3.505218000	0.009952000
H	1.339071000	-2.927609000	0.011282000
O	-3.604113000	4.210340000	-0.004472000
H	-3.163345000	4.431230000	0.830458000
H	-2.937135000	4.371682000	-0.689550000
H	-4.514739000	-3.034956000	0.002448000
H	6.901082000	0.486076000	-0.003232000

Apigeninidin-5OH···H_2O

Energy : -955.4873292 A.U.

NIMAG = 0

Charge = +1 Multiplicity = 1

Stoichiometry $C_{15}H_{13}O_5$

O	6.648030000	0.393444000	-0.065461000
C	5.311897000	0.128630000	-0.031411000
C	4.806984000	-1.181017000	0.001222000
C	3.436770000	-1.388866000	0.025361000
C	4.436596000	1.226490000	-0.031528000
C	3.068755000	1.012963000	-0.006705000
C	2.536877000	-0.298189000	0.019471000
C	1.103689000	-0.514561000	0.031937000
O	0.378634000	0.621183000	0.029381000
C	-0.988361000	0.639019000	0.026752000
C	-1.606995000	1.877566000	0.020048000
C	-3.006544000	1.882426000	0.012905000
O	-3.716070000	3.040411000	0.007214000
C	-3.765917000	0.695173000	0.010319000
C	-3.127024000	-0.533769000	0.017909000
O	-3.782004000	-1.710035000	0.014580000
C	-1.694832000	-0.591845000	0.029174000

C	-0.945766000	-1.779018000	0.037897000
C	0.440170000	-1.743693000	0.040373000
H	5.491633000	-2.024054000	0.004584000
H	3.074014000	-2.409704000	0.048195000
H	4.842058000	2.232459000	-0.054614000
H	2.402736000	1.867360000	-0.010439000
H	-1.031500000	2.796377000	0.019559000
H	-4.848181000	0.757696000	0.003413000
H	-4.774245000	-1.588246000	-0.021036000
H	-1.457505000	-2.734908000	0.040801000
H	1.008088000	-2.662973000	0.043933000
O	-6.435655000	-1.593664000	-0.122207000
H	-6.818528000	-1.222594000	0.687745000
H	-6.708478000	-0.986679000	-0.827569000
H	-3.121657000	3.808433000	0.011325000
H	7.160295000	-0.431752000	-0.076288000

Apigeninidin-4´OH···H_2O

Energy : -955.4870553 A.U.

NIMAG = 0

Charge = +1 Multiplicity = 1

Stoichiometry $C_{15}H_{13}O_5$

O	5.501516000	1.143919000	-0.004048000
C	4.223043000	0.708930000	-0.003163000
C	3.888650000	-0.659265000	-0.011861000
C	2.558631000	-1.045613000	-0.009923000
C	3.201803000	1.678667000	0.006156000
C	1.875671000	1.287265000	0.006749000

C	1.520140000	-0.084285000	-0.000908000
C	0.131896000	-0.485932000	-0.000102000
O	-0.735571000	0.544462000	-0.000945000
C	-2.091887000	0.383835000	-0.000236000
C	-2.862463000	1.534481000	-0.002034000
C	-4.249763000	1.367980000	-0.001333000
O	-5.094669000	2.430652000	-0.003272000
C	-4.850669000	0.092028000	0.001607000
C	-4.058180000	-1.040082000	0.003324000
O	-4.565228000	-2.300290000	0.006121000
C	-2.633635000	-0.926332000	0.002078000
C	-1.735187000	-2.009534000	0.002995000
C	-0.368087000	-1.793641000	0.001498000
H	6.158486000	0.393157000	-0.010993000
H	4.675761000	-1.406291000	-0.019668000
H	2.332721000	-2.105523000	-0.016665000
H	3.470204000	2.729955000	0.012664000
H	1.102168000	2.045945000	0.013864000
H	-2.404306000	2.516895000	-0.004087000
H	-5.932637000	0.010816000	0.002375000
H	-2.117163000	-3.024299000	0.004947000
H	0.315391000	-2.630970000	0.002484000
O	7.422601000	-0.725846000	-0.073209000
H	7.951750000	-0.691662000	0.738529000
H	7.050311000	-1.620969000	-0.091745000
H	-4.595245000	3.264369000	-0.004923000
H	-5.536422000	-2.275608000	0.006969000

Corresponding neutral quinoidal base (Apigeninidin -7OH···H_2O)

Energy : -955.0308242 A.U.

NIMAG = 0

Charge = 0 Multiplicity = 1

Stoichiometry $C_{15}H_{12}O_5$

O	6.516751000	-1.187912000	0.012660000
C	5.235695000	-0.708106000	0.009239000
C	4.955505000	0.665838000	0.004541000
C	3.637384000	1.100272000	0.000687000
C	4.190894000	-1.642307000	0.010491000
C	2.875057000	-1.201140000	0.006734000
C	2.568413000	0.177851000	0.001852000
C	1.184045000	0.634725000	-0.001122000
O	0.283192000	-0.376854000	-0.013662000
C	-1.076278000	-0.173776000	-0.016435000
C	-1.886838000	-1.279573000	-0.028606000
C	-3.308788000	-1.100573000	-0.030690000
O	-4.106563000	-2.107104000	-0.041363000
C	-3.824003000	0.250677000	-0.019980000
C	-2.990477000	1.338030000	-0.008718000
O	-3.439309000	2.629388000	0.001207000
C	-1.562216000	1.174523000	-0.006330000
C	-0.638000000	2.210491000	0.005516000
C	0.737696000	1.944377000	0.008306000
H	5.772072000	1.382261000	0.003370000
H	3.448624000	2.167614000	-0.003937000
H	4.420486000	-2.702795000	0.014600000
H	2.076208000	-1.932971000	0.008223000
H	-1.466818000	-2.279034000	-0.035920000
H	-4.901128000	0.391596000	-0.021730000
H	-0.979925000	3.239554000	0.013480000
H	1.447257000	2.759651000	0.019031000
O	-6.797716000	-1.809482000	0.052040000
H	-6.989546000	-0.887182000	0.272682000
H	-5.805591000	-1.863423000	0.022575000
H	7.149309000	-0.450989000	0.009966000
H	-4.410320000	2.643577000	-0.002297000

Corresponding neutral quinoidal base (Apigeninidin -5OH···H_2O)

Energy : -955.0301973 A.U.

NIMAG = 0

Charge = 0 Multiplicity = 1

Stoichiometry $C_{15}H_{12}O_5$

O	6.628247000	0.400343000	0.000025000
C	5.287667000	0.132391000	-0.000004000
C	4.785063000	-1.177260000	-0.000005000
C	3.413373000	-1.388476000	-0.000015000
C	4.409981000	1.225762000	-0.000032000
C	3.040115000	1.007652000	-0.000044000
C	2.511000000	-0.302366000	-0.000030000
C	1.071211000	-0.524635000	-0.000018000
O	0.347332000	0.617647000	-0.000016000
C	-1.025668000	0.634696000	0.000005000
C	-1.645611000	1.864530000	-0.000004000
C	-3.058002000	1.856924000	0.000020000
O	-3.751645000	3.034694000	0.000011000
C	-3.815815000	0.687152000	0.000052000
C	-3.200286000	-0.587424000	0.000060000
O	-3.860548000	-1.691660000	0.000087000
C	-1.733114000	-0.604647000	0.000033000
C	-0.984379000	-1.775958000	0.000025000
C	0.413705000	-1.744975000	-0.000004000
H	5.470665000	-2.019746000	0.000008000
H	3.052501000	-2.410481000	-0.000009000
H	4.811263000	2.233848000	-0.000038000
H	2.372676000	1.861000000	-0.000062000
H	-1.080153000	2.789634000	-0.000027000
H	-4.898657000	0.755541000	0.000076000

H	-1.497963000	-2.731745000	0.000042000
H	0.979845000	-2.665623000	-0.000010000
O	-6.562420000	-1.685837000	-0.000114000
H	-6.863064000	-0.766259000	-0.000317000
H	-5.568939000	-1.630646000	0.000068000
H	7.138819000	-0.425521000	0.000094000
H	-3.140157000	3.788308000	-0.000007000

Corresponding neutral quinoidal base (Apigeninidin -4´OH···H_2O)

Energy : -955.0280614 A.U.

NIMAG = 0

Charge = 0 Multiplicity = 1

Stoichiometry $C_{15}H_{12}O_5$

O	5.689469000	-0.227823000	-0.054146000
C	4.409401000	-0.355696000	-0.046262000
C	3.784545000	-1.652773000	-0.056567000
C	2.418786000	-1.794576000	-0.043042000
C	3.527057000	0.783513000	-0.026794000
C	2.162177000	0.633939000	-0.016580000
C	1.552987000	-0.658226000	-0.021557000
C	0.140227000	-0.805821000	-0.005135000
O	-0.555131000	0.361208000	-0.020366000
C	-1.924158000	0.420211000	-0.009556000
C	-2.497713000	1.683727000	-0.031017000
C	-3.893857000	1.750147000	-0.022141000
O	-4.558724000	2.940396000	-0.044571000
C	-4.693529000	0.592676000	0.010684000
C	-4.090147000	-0.653814000	0.033064000
O	-4.802742000	-1.815009000	0.066202000
C	-2.673003000	-0.776457000	0.021996000
C	-1.961057000	-2.004086000	0.041553000

C	-0.588108000	-2.018451000	0.027066000
H	4.427271000	-2.528320000	-0.075092000
H	2.003178000	-2.796363000	-0.053404000
H	3.970031000	1.775369000	-0.019854000
H	1.532964000	1.517056000	-0.000828000
H	-1.883031000	2.576704000	-0.054864000
H	-5.774625000	0.686204000	0.017038000
H	-2.509536000	-2.938885000	0.068091000
H	-0.053278000	-2.957566000	0.043946000
O	6.893170000	2.185371000	0.135944000
H	6.397118000	1.325941000	0.063831000
H	6.206374000	2.862842000	0.209488000
H	-3.932179000	3.681781000	-0.071995000
H	-5.754264000	-1.623777000	0.074874000

Absolute Energies

Table S 1 Absolute energies (in A.U) of each optimized geometry at fixed R distance in model molecules. R corresponds to O–H distance in the discrete H_2O molecule hydrogen bonded to the carbonyl group of the quinoidal base.

7-OH		5-OH		4′-OH	
R (Å)	E (A.U)	R (Å)	E (A.U)	R (Å)	E (A.U)
0.99	-1033.626820	0.99	-1033.625933	1.00	-1033.623361
1.07	-1033.624134	1.07	-1033.623324	1.07	-1033.620825
1.14	-1033.619008	1.14	-1033.618346	1.14	-1033.615987
1.22	-1033.614234	1.22	-1033.613704	1.22	-1033.611557
1.29	-1033.610689	1.29	-1033.610234	1.29	-1033.608375
1.37	-1033.608150	1.37	-1033.607715	1.36	-1033.606141
1.44	-1033.606151	1.44	-1033.605704	1.44	-1033.604359
1.52	-1033.604336	1.51	-1033.603877	1.51	-1033.602721
1.59	-1033.602563	1.59	-1033.602091	1.59	-1033.601101
1.67	-1033.600803	1.66	-1033.600322	1.66	-1033.599478
1.74	-1033.599092	1.74	-1033.598596	1.73	-1033.597870

Table S 2 Absolute energies (in A.U) of each optimized geometry at fixed R distance in Apigeninidin. R corresponds to O–H distance in the discrete H_2O molecule hydrogen bonded to the carbonyl group of the quinoidal base

7-OH		5-OH		4′-OH	
R (Å)	E (A.U)	R (Å)	E (A.U)	R (Å)	E (A.U)
0.99	-955.030824	1.00	-955.030197	0.99	-955.028061
1.07	-955.028172	1.07	-955.027644	1.07	-955.025508

1.14	-955.023041	1.14	-955.022788	1.14	-955.020637
1.22	-955.018305	1.22	-955.018251	1.22	-955.016161
1.29	-955.014834	1.29	-955.014862	1.29	-955.012937
1.37	-955.012319	1.36	-955.012384	1.36	-955.010667
1.44	-955.010297	1.44	-955.010398	1.44	-955.008857
1.52	-955.008447	1.51	-955.008584	1.51	-955.007193
1.59	-955.006640	1.59	-955.006805	1.59	-955.005545
1.67	-955.004849	1.66	-955.005048	1.66	-955.003904
1.74	-955.003099	1.73	-955.003335	1.73	-955.002281

References

(1) L. Cruz, N. B., N. Mateus, V. de Freitas, F, Pina. Natural and Synthetic Flavylium-Based Dyes: The Chemistry Behind the Color. *Chem. Rev.* 2022**, 122**, 1416-1481.

(2) R. Brouillard, J. E. Dubois. Mechanism of Structural Transformations of Anthocyanins in Acidic Media. *J. Am. Chem. Soc.* 1977**, 99**, 1359-1364.

(3) F. Pina, M. Maestri, V. Balzani. Photochromic Flavylium Compounds as Multistate/Multifunction Molecular-Level Systems. *Chem. Commun.* 1999, 107-114.

(4) F. Pina, L. Benedito,M.J. Melo, A. J. Parola, A. Bernardo, Photochemistry of 3,4'-Dimethoxy-7-Hydroxyflavylium Chloride - Photochromism and Excited-State Proton Transfer. *Journal of the Chemical Society-Faraday Transactions* 1996**, 92**, 1693-1699.

(5) F. Pina, Anthocyanins and Related Compounds. Detecting the Change of Regime between Rate Control by Hydration or by Tautomerization. *Dyes Pigm.* 2014**, 102**, 308-314.

(6) R. A. McClelland, S. Gedge, S. Hydration of the Flavylium Ion *J. Am. Chem. Soc.* 1980**, 102**, 5838-5848.

(7) H. Mahmoodi, N. Basilio, P. S. Branco, J. C. Lima, F. Pina, Calculation of the Absorption Spectra of Various Anthocyanin Species in an Acidic Medium Using Stopped-Flow Spectroscopy. *J. Org. Chem.* 2025**, 90**, 14285-14290.

(8) J. Mendoza, N. Basilio, V. de Freitas, F. Pina, New Procedure to Calculate All Equilibrium Constants in Flavylium Compounds: Application to the Copigmentation of Anthocyanins. *ACS Omega* 2019**, 4**, 12058-12070.

(9) V. Navarkhele, A. Navarkhele, Static Dielectric Constants, Densities, Refractive Indices and Related Properties of Binary Mixtures at Various Temperatures Under Atmospheric Pressure, I*nt. J. Thermodyn.* 2022, **25**, 1-10.
(10) S. Mirzaei, M. V. Ivanov, Q.K. Timerghazin, Improving Performance of the SMD Solvation Model: Bondi Radii Improve Predicted Aqueous Solvation Free Energies of Ions and pK_a Values of Thiols, *J. Phys. Chem. A.* 2019, **123**, 9498-9504.
(11) S. Pezzola, M. Venanzi, P. Galloni, V. Conte, F. Sabuzi, Easy to Use DFT Approach for Computational *p*Ka Determination of Carboxylic Acids, *Chem. Eur. J.* 2024, **30**, e202303167 (1 of 7)
(12) A. A. Freitas; K. Shimizu ; L. G. Dias; F. H. Quina, A computational study of substituted flavylium salts and their quinonoidal conjugate-bases: S_0® S_1 electronic transition, absolute pK_a and reduction potential calculations by DFT and semiempirical methods, *J. Braz. Chem. Soc.* 18, 2007
(13) Jing Cui, F. Siddique, R. Nieman, G. T. M. Silva, F. H. Quina, A. J. A. Aquino, Quantum chemical investigation of the ground- and excited-state acidities of a dihydroxyfuranoflavylium cation, *Theor. Chem. Acc.*, 2021, 140:90
